# The Parallel Presentation of Two Functional CTL Epitopes Derived from the O and Asia 1 Serotypes of Foot-and-Mouth Disease Virus and Swine SLA-2*HB01: Implications for Universal Vaccine Development

**DOI:** 10.3390/cells11244017

**Published:** 2022-12-12

**Authors:** Lei Feng, Yong-Yu Gao, Mingwei Sun, Zi-Bin Li, Qiang Zhang, Jie Yang, Cui Qiao, Hang Jin, Hong-Sheng Feng, Yu-Han Xian, Jianxun Qi, George F. Gao, William J. Liu, Feng-Shan Gao

**Affiliations:** 1 Department of Bioengineering, College of Life and Health, Dalian University, Dalian 116622, China; 2 CAS Key Laboratory of Pathogen Microbiology and Immunology, Institute of Microbiology, Chinese Academy of Sciences (CAS), Beijing 100101, China; 3 College of Animal Medicine, Jilin Agricultural University, Changchun 130118, China; 4 Department of Microbiology and Immunology, College of Veterinary Medicine, China Agricultural University, Beijing 100094, China; 5 State Key Laboratory of Veterinary Etiological Biology, Lanzhou Veterinary Research Institute, Chinese Academy of Agricultural Sciences, Lanzhou 730046, China; 6 NHC Key Laboratory of Biosafety, Research Unit of Adaptive Evolution and Control of Emerging Viruses, Chinese Academy of Medical Sciences, National Institute for Viral Disease Control and Prevention, Chinese Center for Disease Control and Prevention, Beijing 102206, China

**Keywords:** SLA-2, foot-and-mouth disease virus, crystal, peptide, epitope, CTL, universal vaccine candidate

## Abstract

Foot-and-mouth disease virus (FMDV) poses a significant threat to the livestock industry. Through their recognition of the conserved epitopes presented by the swine leukocyte antigen (SLA), T cells play a pivotal role in the antiviral immunity of pigs. Herein, based on the peptide binding motif of SLA-2*HB01, from an original SLA-2 allele, a series of functional T-cell epitopes derived from the dominant antigen VP1 of FMDV with high binding capacity to SLA-2 were identified. Two parallel peptides, Hu64 and As64, from the O and Asia I serotypes, respectively, were both crystallized with SLA-2*HB01. Compared to SLA-1 and SLA-3, the SLA-2 structures showed the flexibility of residues in the P4, P6, and P8 positions and in their potential interface with TCR. Notably, the peptides Hu64 and As64 adopted quite similar overall conformation when bound to SLA-2*HB01. Hu64 has two different conformations, a more stable ‘chair’ conformation and an unstable ‘boat’ conformation observed in the two molecules of one asymmetric unit, whereas only a single ‘chair’ conformation was observed for As64. Both Hu64 and As64 could induce similar dominant T-cell activities. Our interdisciplinary study establishes a basis for the in-depth interpretation of the peptide presentation of SLA-I, which can be used toward the development of universal vaccines.

## 1. Introduction

The foot-and-mouth disease virus (FMDV) belongs to the *Aphthovirus* genus of *Picornaviridae*, a family of small, icosahedral, non-enveloped, single-stranded RNA viruses [1]. The virus causes an acute, febrifacient, and highly contagious viral disease unique to cloven-hooved animals and is a major threat to swine and other livestock and their associated industries. There are seven serotypes in FMDV: A, O, C, Asia 1, SAT1, SAT2, and SAT3. However, infection with any one of these serotypes does not produce significant cross-immunity protection against the other serotypes [2]. The P1 gene in the FMDV genome encodes the viral capsid proteins. VP1 harbors the pivotal antigenic variation site, most of which is exposed on the virus protein surface, which mediates the viral infection of cells [3]. Variability between FMDV strains is usually caused by variances in the VP1 gene. In FMD-endemic areas, vaccination is used as a preventive method. Because of variations in serotype prevalence in different areas, the most commonly used vaccines are against serotypes O and A [4,5]. Vaccines specific to the Asia 1 or SATs serotypes have also been used for decades [6,7,8]. Conventional FMD vaccines based on chemically inactivated FMDVs that are formulated with an adjuvant mainly induce hosts to produce humoral immunity with specific neutralizing antibodies in order to protect animals [9]. However, this provides only short-term and serotype-specific protection [10]. In addition, other concerns have been raised recently, including the risk of virus release during vaccine production, thermal instability, and the fact that these vaccines contain traces of non-structural proteins (NSP), making it difficult to distinguish between vaccinated and infected animals when using currently approved assays [11,12,13]. Therefore, other alternative and safe FMDV vaccines should be developed. For instance, vaccines containing multi-epitopes might overcome the above-described shortcomings of conventional FMD vaccines. Cubillos reported a specific T cell epitope that can increase the protection conferred against foot-and-mouth disease virus in pigs by a linear peptide containing an immunodominant B cell site [10], but the T cell epitope [3A (21–35)] should be a T helper (Th) epitope that is recognized by CD4^+^ T cells [14,15]. More attention should be paid to cellular immunity to the CD8^+^ cytotoxic T lymphocyte (CTL)-mediated cellular immune response, which can kill virus-infected target cells in order to eliminate the virus in the host’s cells [16,17]. CTL-mediated cellular immunity is induced by CTL epitopes, which are complexed with major histocompatibility complex I and presented by antigen-presenting cells (APCs) [18,19].

MHC class I molecules are particularly responsible for CD8^+^ cytotoxic T lymphocyte (CTL) cellular immunity due to their ability to bind to antigenic peptides based on their special three-dimensional structure [20,21]. Bjorkman et al. [22] first determined the three-dimensional structure of human MHC class I, i.e., human leukocyte antigen (HLA)-A2 molecules. It was shown that HLA-A2 is constituted by a heavy chain of α and a light chain of β_2_m. The heavy chain of α is divided into an extracellular region, a transmembrane region, and an intracellular region. The extracellular region consists of three domains: α1, α2, and α3. The β_2_m binds non-covalently to the α3 domain of MHC class I molecule and lacks a transmembrane region. The α1 and α2 domains of the molecule form the peptide-binding groove (PBG) with six pockets (A–F) [23], which together accommodate 8-11 amino acids from antigenic peptides [24,25]. The binding of antigenic peptides to the PBG of MHC I results in recognition by the T-cell receptor (TCR) in order to induce CD8^+^ cytotoxic T lymphocyte (CTL)-mediated cellular immunity [26,27]. In recent years, the structures of MHC I molecules of more species have been determined [28,29], which has greatly promoted the development of comparative immunology research and veterinary vaccines.

Swine MHC is also known as swine leukocyte antigen (SLA). SLA class I also contains a heavy chain and a light chain. The heavy chain is formed from the products encoded by three genes, SLA-1, SLA-2, and SLA-3, which all display polymorphisms. Among these three genes, the SLA-2 locus is easily distinguished from SLA-1 and SLA-3 by its longer signal peptide. A further dissimilarity to SLA-1 and SLA-3 is in the identity of the three amino acid residues at the start of the signal peptide [30]. The light chain is encoded by swine β_2_m (sβ_2_m). In the endoplasmic reticulum, the SLA-I heavy chain, antigenic peptides, and sβ_2_m noncovalently associate into an SLA-I–peptide–sβ_2_m complex. After processing in the Golgi apparatus, the SLA-I–peptide–sβ_2_m complex is transported to the surfaces of antigen presenting cells (APCs), where it can interact with the T-cell receptors (TCRs) of CD8^+^ T lymphocytes to induce a specific cellular immune response. The elaborate crystal structure of SLA-I has been reported for SLA-1*0401 and SLA-3*hs0202 [31,32]. It was shown that the PBG of SLA-3 had different peptide-binding preferences to those of SLA-1, especially in the P2, P3, and P9 positions. Up to now, the three-dimensional structures of SLA-2 molecules have been identified, and the crystal of SLA-2*HB01 has been described, albeit without structural information [33,34,35]. Specifically, there has been insufficient investigation into the structural characteristics of SLA-2 molecules.

In this study, we first designed a series of peptides derived from three serotypes of FMDV, O, A, and Asia 1; then, crystallization was carried out for SLA-2*HB01 and sβ_2_m complexed with these putative CTL peptide epitopes. The structural distinctions between SLA-2 and the other two SLA molecules are reported. The structural and functional data demonstrate the similar overall conformation of the two peptides Hu64 and As64 from FMDV O and Asia 1 with CTL activities. As a result, our research provides detailed structural data about SLA-2 molecules and supplies details of important functional CTL epitopes that may contribute to the development of an FMDV epitope-based universal vaccine.

## 2. Materials and Methods

### 2.1. Animals, Genes, and Viruses

Twelve 1-month-old specific-pathogen-free (SPF) Hebao pigs were bred as experimental animals. The Hebao pig has been inbred for more than 300 years in an enclosed mountainous terrain, which means that the breeding of these pigs evolved independently for a long period of time. The genetic characteristics of SLA-2*HB01 (AB602431) in Hebao pigs were reported previously [36]. Three different serotypes of foot-and-mouth disease virus strains, including O-Tibet/CHA/99 (O-HuBHK99), A-HuBWH (1) 2009, and Asia 1/Jiangsu/China/2005, were authorized by the National FMD Reference Laboratory of China in the Lanzhou Veterinary Research Institute (LVRI) of the Chinese Academy of Agricultural Sciences (CAAS) for challenging animals. The pig median infected dose (PID_50_) of the three virus strains in suckling mice was evaluated as 5.0 log10/2 mL using the method described by Li et al. [37]. The same batch of viruses passaged in suckling mice were used in this study.

### 2.2. Epitope Prediction and Peptide Synthesis

A total of nine peptides were designed for use as epitopes in the experiments (Table 1). Based on the antigen (VP1 structural protein and 3D RNA polymerase) of foot-and-mouth disease virus strains, potential SLA-2-restricted CD8^+^ T-cell epitopes were predicted using the NetMHCpan-2.8 tool (http://www.cbs.dtu.dk/services/NetMHCpan, accessed on 9 May 2014). These peptides (candidate epitopes) were synthesized by ChinaPeptides Co., Ltd. (Shanghai, China). The purities of the peptides were over 90%, as assessed by LC–MS/MS.

### 2.3. Crystallization of SLA-2*HB01 and sβ_2_m Complexed with Peptides

The preparation, refolding, and crystallization of SLA-2*HB01 and sβ_2_m proteins complexed with peptides were performed as previously reported [35,39]. In this research, additional peptides (shown in Table 1) were used in refolding and crystallization with SLA-2*HB01 and sβ_2_m proteins. Two peptides, Hu64, which is derived from the O serotype of FMDV Tibet/CHA/99 (HuBHK99), and As64, from 1/Jiangsu/China/2005, were crystallized with SLA-2*HB01 and sβ_2_m, and their crystal data were collected as previously reported [35,39].

### 2.4. Determination and Refinement of Structures

The structures of SLA-2*HB01–Hu64–sβ_2_m and SLA-2*HB01–As64–sβ_2_m were determined by molecular replacement using the MOLREP program, with HLA-A*1101 (PDB code: 1Q94) as the search model. Extensive model building was manually performed using COOT [40], and restrained refinement was performed using REFMAC5. Further rounds of refinement were performed using the phenix.refine program implemented in the PHENIX package [41] with isotropic ADP refinement and bulk solvent modeling, which improved the R and R-free factors from 0.194 and 0.209 to 0.151 and 0.177, respectively. The stereochemical quality of the final model was assessed using the pyMOL program [42]. The electron density of the two crystal structures was distributed as part of the CCP4 suite v.7.0 [43,44] and visualized in pyMOL.

### 2.5. Analysis of TCR Contact with CTL Epitopes in Different SLA Class I Crystals

Based on the protein sequences for the swine TCR α chain and β chain in the National Center for Biotechnology Information database (https://www.ncbi.nlm.nih.gov/protein/, accessed on 11 July 2019) and for human TCR in the PDB database, SWISS-MODEL was used for homology-based modeling of the 3D structure of the swine TCR α chain and β chain. PDB data from swine MHC class I crystals and a modeled 3D structure of the swine TCR α chain and β chain were input into the ZDOCK server (http://zdock.umassmed.edu/, accessed on 20 July 2019) to assemble the complex constituted by swine MHC class I and TCR. The protein–protein interactions were analyzed using pyMOL.

### 2.6. Virus Challenge and Infection

The challenge experiments were carried out in the third-level biological safety protection laboratory (P3) of the State Key Laboratory of Veterinary Etiological Biology in Lanzhou Veterinary Research Institute at the Chinese Academy of Agricultural Sciences. The experimental animals were divided into four groups, including a control group and three challenged groups, O, A, and Asia 1. Each group comprised three weaned pigs aged around two months. The pigs in each group were housed in one independent room. The pigs in the challenged groups (O, A, and Asia 1) were challenged in the neck, behind the ear, with one of the field isolated strains Tibet/CHA/99 (HuBHK99), A-HuBWH (1) 2009, and 1/Jiangsu/China/2005, according to their respective group. The pigs in the control group were inoculated using the same dose of PBS. The schedule used for the virus challenge of the pigs is given in Table 2. Daily observations were then conducted and clinical signs were recorded for each pig for 20 days post-challenge as described by Rieder et al. [45]. Anti-clotted blood samples were collected at 15 and 20 days post-challenge (dpc).

All the animal experiments were performed following the management guidelines of the Gansu Ethical Review Committee (License no. SYXK[GAN]2014-003).

### 2.7. ELISPOT Assays

Peripheral blood mononuclear cells (PBMCs) from pigs infected with the corresponding virus strain were separated using a Swine Lymphocyte Separation Kit (DAKEWE Company, Shanghai, China) 20 dpc. The antigen-specific T-cell responses were detected using IFN-γ ELISPOT assays as previously described [38] but with slight modifications. Briefly, the assays were performed in pre-coated 96-well plates (MABTECH Company, Stockholm, Sweden). A total of 2.5 × 10^5^ PBMCs in 100 μL RPMI 1640 medium supplemented with 10% FBS were seeded in each well of the 96-well ELISPOT plates. To stimulate the Ag-specific T-cell producing IFN-γ, the individual peptide (with a final concentration of 10 μg/mL) corresponding to the serotype of PBMCs was diluted in 100 μL RPMI 1640 supplemented with 10% FBS added to each well, and then incubated at 37 °C with 5% CO_2_ for 16 h. Con A was added as a positive control for nonspecific stimulation. Cells incubated without the stimulator functioned as the negative control, producing fewer than five spots in 90% of the experiments. A 9-mer peptide (QPNEAIRSL), as previously reported, was used as a negative control peptide [46]. All of the wells were duplicated to minimize possible discrepancies. Then, all the medium-containing cells and peptides in each well were removed and the plates were processed according to the manufacturer’s instructions. Finally, the colored spots were counted with an ImmunoSpot^®^ Analyzer and analyzed using the ImmunoSpot 5.0 Academic software (both from Cellular Technology Ltd., Bonn, Germany). Mean data were calculated and expressed as spot-forming cells [SFCs]/2.5 × 10^5^ PBMCs. ELISPOT analysis was performed at least three times per peptide.

### 2.8. Accession Numbers for Protein Structures

The crystal structures of SLA-2*HB01–Hu64–sβ_2_m and SLA-2*HB01–As64–sβ_2_m were deposited into the Protein Data Bank (https://deposit-pdbj.wwpdb.org/deposition, accessed on 31 August 2022) with accession numbers 8GQW and 8GQV, respectively.

## 3. Results

### 3.1. Overall Structures of the SLA-2-HB01 Complexes and the Conformations and Flexibilities of the SLA-I-Binding Peptides

The crystals of SLA-2*HB01–Hu64–sβ_2_m and SLA-2*HB01–As64–sβ_2_m were all found to belong to space group P2_1_2_1_2_1_; all of the X-ray diffraction data for both crystals are presented in previous articles [35,39].

**Figure 1:**
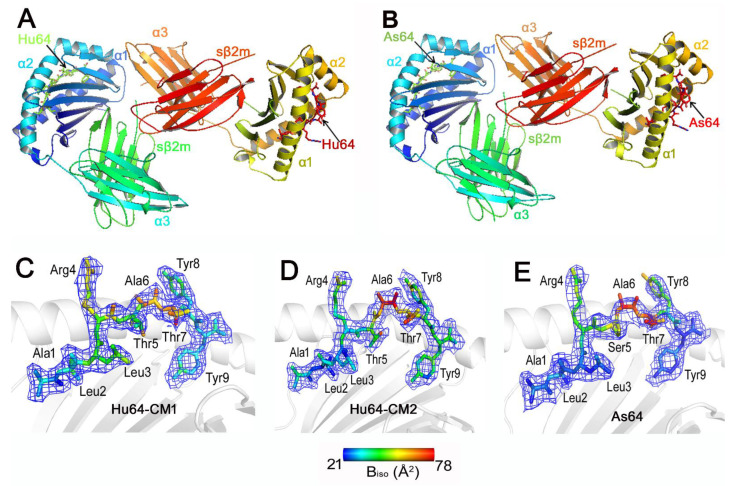
Analysis of the overall structure of SLA-2*HB01 and analysis of the conformations and flexibilities of peptides bound to SLA-2*HB01. The structure of (**A**) SLA-2*HB01–Hu64–sβ_2_m; (**B**) SLA-2*HB01–As64–sβ_2_m. The conformation when peptide (**C**) Hu64-CM1, (**D**) Hu64-CM2, or (**E**) As64 are bound to the peptide-binding groove of SLA-2*HB01, presented with electron density at the 1.0-σ contour level. The flexibility of amino acids in different peptides are shown in different colors according to isotropic B factors.

The structures of the swine MHC I molecule SLA-2*HB01 carrying FMDV-derived immunogenic peptides (Hu64) were determined to a resolution of 2.5 Å. The crystal was estimated to contain two SLA-2*HB01–Hu64–sβ_2_m molecules in the asymmetric unit (Figure 1A). The resolution of the SLA-2*HB01–As64–sβ_2_m crystal was 2.4 Å, also with two molecules in the asymmetric unit (Figure 1B). The overall structure of SLA-2*HB01 retains the characteristics of MHC I molecules common in other mammals. The extracellular region of the SLA-2*HB01 heavy chain folds into three different domains: the α1, α2, and α3 domains. Among these, the α1 and α2 domains form a typical peptide-binding groove, which contains two α-helices and eight β-sheets, and the Hu64 and As64 peptides are embedded in the groove. The α3 domains of SLA-2*HB01 and sβ_2_m display typical immunoglobulin superfamily domains and underpin the α1 and α2 domains. The light chain of sβ_2_m is noncovalently ligated to the α3 domain of SLA-2*HB01.

The electron densities of the peptides in the structures of both SLA-2*HB01 complexes are shown in detail in Figure 1C–E. In summary, Hu64 and As64 are all arched, and their N and C termini display tight contacts with the groove residues of SLA-2*HB01. Interestingly, it was found that Hu64 displayed two different electron density states, indicating that there might be two conformations in the refolding of Hu64-binding SLA-2*HB01 molecules. The first conformation (CM1) of Hu64 is tighter than that of the second conformation of Hu64 (CM2) in terms of electron density. In addition, it seems that, according to the isotropic B factors, the conformation of residues from P6 to P9 in CM2 is more flexible than that of CM1. Therefore, CM1 should be more stable than CM2. The N and C termini both appeared to have more stable conformation in As64 than in Hu64-CM1, whereas the middle positions (P5 to P7) should also be more flexible in As64 than in Hu64-CM1. Because the N and C termini of peptides mainly determine the binding capacity with MHC class I molecules, it is speculated that As64 might be bound with SLA-2*HB01 in a slightly more stable way than Hu64 overall. Meanwhile, As64 might be more flexible in its contact with TCR than Hu64, according to previously reported findings [47]. When either Hu64-CM1 or As64 is bound to SLA-2*HB01 molecules, the P5 (Thr/Ser) is buried within the grooves, whereas P4 (Arg), P6 (Ala) and P8-Tyr are almost entirely exposed to the solvent; this likely plays a dominant role in their recognition by TCR.

**Figure 2:**
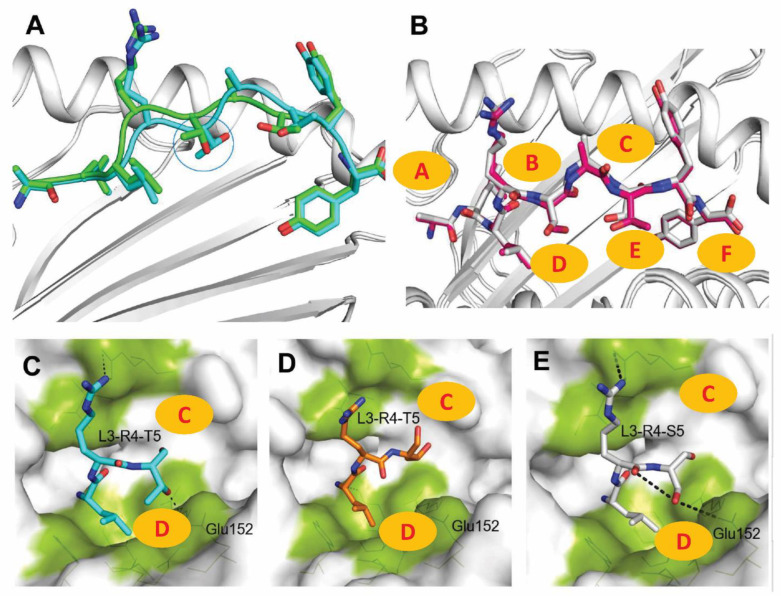
Analysis and comparison of the two conformations of the crystals of SLA-2*HB01–Hu64–sβ_2_m and SLA–2*HB01–As64–sβ_2_m. The two conformations of the crystals of (**A**) SLA-2*HB01–Hu64–sβ_2_m and (**B**) SLA-2*HB01–As64–sβ_2_m. The marine circle in (**A**) represents P5 of the two Hu64 refolded conformations. The refolding state of peptide Hu64 in the peptide-binding groove of SLA-2*HB01–Hu64–sβ_2_m in (**C**) crystal conformation 1 (CM1) and (**D**) crystal conformation 2 (CM2). (**E**) The refolding state of peptide As64 in the peptide-binding groove of the SLA-2*HB01–As64–sβ_2_m crystal. Areas marked A–F in highlighted brown indicate the different pockets in the peptide-binding groove. The dotted lines in (**C**,**E**) indicate the key hydrogen bonds between atoms of the peptides and atoms of the main chain residues.

Through analysis of the two conformations of the crystals of SLA-2*HB01–Hu64–sβ_2_m and SLA-2*HB01–As64–sβ_2_m, it was shown that for the Hu64 peptides, the two conformations of the crystals display different folding states in amino acids, such as in position five (P5) (Figure 2A). However, the crystals of SLA-2*HB01–As64 showed no clear difference in peptide folding in the two conformations (Figure 2B). Through further comparison of the peptides’ refolding states between the different crystals and their conformations, it was shown that the P5 (Thr) for peptide Hu64 in the crystals of SLA-2*HB01–Hu64–sβ_2_m was altered at different conformation states. In conformation 1 (CM1) (Figure 2C), the P5-Thr formed a hydrogen bond with the Glu 152 in the side chain of the peptide-binding groove, whereas this did not occur in conformation 2 (CM2) (Figure 2D). By way of analogy to chemistry, the CM1 adopts a ‘chair’ conformation, indicating a more stable conformation, whereas CM2 resembles a ‘boat’ conformation, indicating an unstable conformation [48]. In addition, P5-Thr serves as an anchor amino acid in CM1 but not in CM2. Accordingly, it is supposed that CM1 of the SLA-2*HB01–Hu64 crystal should be more stable. For crystals of SLA-2*HB01–As64, the conformation is similar to the CM1 of the SLA-2*HB01–Hu64–sβ_2_m crystal, where P5-Ser also serves as an anchor amino acid (Figure 2E). Comprehensive analysis of the mutual interaction indicates that the binding state of As64 in the SLA-2*HB01–As64–sβ_2_m crystal should be more stable.

By further comparing between SLA-2*HB01–Hu64–sβ_2_m-CM1 and SLA-2*HB01–As64–sβ_2_m on the basis of peptide hydrogen bonding and van der Waals interactions, it was shown that the main difference existed in the P5 residue, i.e., the former P5 (Thr5) can form hydrogen bonds with the residue 152 of the main chain, whereas the later P5 (Ser) cannot form hydrogen bonds with any residues of the main chain (Table 3). For other peptide sites, the type of hydrogen bond and the binding residues, even on the atomic level, were all consistent with each other except when the distance of the hydrogen bond between two crystals was different. However, there were many different residues for which there was van der Waals contact between the two crystals, such as P1 (Ala)–Leu5, P5 (Thr)–Arg97, and P7 (Thr)–Val150 from SLA-2*HB01–Hu64–sβ_2_m-CM1, and P1 (Ala)-Arg62, P2 (Leu)–Arg163, P3 (Leu)-Arg97, P6 (Ala)–Ser5, P6(Ala)–Thr7, P7 (Thr)–Asp77, and P9 (Tyr)–Trp147 from SLA-2*HB01–As64–sβ_2_m.

### 3.2. Comparison of the Peptide-Binding Interfaces and the Pockets of SLA-2*HB01, SLA-1*0401, and SLA-3*hs0202

In order to learn more about the differences in the peptide binding interfaces and pockets between SLA-2*HB01 and other SLA-I molecules, we compared the amino acid sequence of SLA-2*HB01 with those of SLA-1*0401 and SLA-3*hs0202. It was shown that the homologies were 83.3 and 86.7 % in the α1 domain, respectively; 83.3 and 80.0 % in the α2 domain; and 99.0 and 99.0 % in the α3 domain. As expected, most of the polymorphic residues are located in the α1 and α2 domains. However, the secondary structure elements among SLA-2*HB01, SLA-1*0401, and SLA-3*hs0202 were nearly completely consistent (Figure S1).

**Figure 3:**
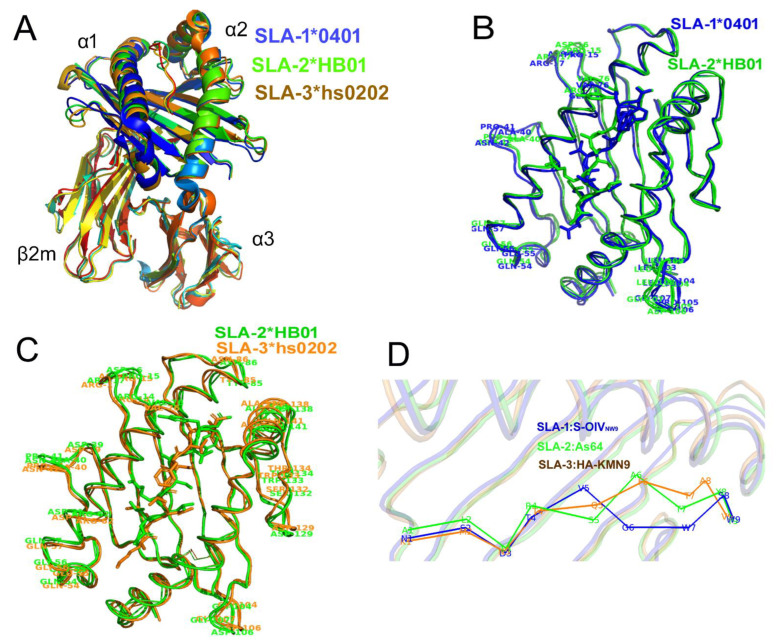
A comparison of the crystal structures of SLA-2*HB01, SLA-1*0401, and SLA-3*hs0202. (**A**) The superposition of the overall structures of SLA-2*HB01, SLA-1*0401, and SLA-3*hs0202, which are all displayed as a cartoon model, with a heavy chain comprising the α1, α2, and α3 domains and a light chain sβ_2_m. (**B**) Aligning the peptide-binding grooves of SLA-2*HB01 and SLA-1*0401. The peptide S-OIV_NW9_ bound to SLA-1*0401 and the peptide As64 bound to SLA-2*HB01 are shown by blue and green sticks, respectively. The peptide-binding grooves from SLA-1*0401 and SLA-2*HB01 are respectively shown by blue and green loops. The labeled amino acids are shown in the same color as their chains. (**C**) Aligning the peptide-binding grooves of SLA-2*HB01 and SLA-3*hs0202. The peptide As64 bound to SLA-2*HB01 and the peptide HA kmN9 bound to SLA-3*hs0202 are shown by green and orange sticks, respectively. The peptide-binding groove from SLA-2*HB01 and the peptide-binding groove from SLA-3*hs0202 are respectively shown by green and orange loops. The labeled amino acids are shown in the same color as their chains. (**D**) Comparing the peptide refolding of SLA-1*0401, SLA-2*HB01, and SLA-3*hs0202. The peptides are all shown in cartoon style and labeled in different colors. The blue color indicates S-OIV_NW9_ from SLA-1*0401; the green indicates As64 from SLA-2*HB01; and the orange indicates HA kmN9 from SLA-3*hs0202. The amino acids (one letter) are labeled in the same color as in their cartoon models.

By comparing the integral crystal structures, it was found that SLA-2*HB01 is similar to SLA-1*0401 and SLA-3*hs0202 (Figure 3A). The superposition of SLA-2*HB01–Hu64–sβ_2_m-CM1 onto the previously determined structure of SLA-1*0401–S-OIV_NW9_ generated a root mean square deviation (RMSD) of 0.846, whereas the RMSD between SLA-2*HB01–As64–sβ_2_m and SLA-1*0401–S-OIV_NW9_ was 0.808. Compared to SLA-1*0401–Ebola_AY9_, the RMSD values for SLA-2*HB01–Hu64–sβ_2_m-CM1 and SLA-2*HB01–As64–sβ_2_m are 0.780 and 0.733, respectively. Compared to SLA-3*hs0202–HA kmN9, the RMSD values for SLA-2*HB01–Hu64–sβ_2_m-CM1 and SLA-2*HB01–As64–sβ_2_m are 0.891 and 0.902, respectively. When we superimposed the α1 and α2 domains of SLA-2*HB01–As64–sβ_2_m, SLA-1*0401–S-OIV_NW9_, and SLA-3*hs0202–HA kmN9, we found the most distinct portion of the SLA-1*0401 and SLA-2*HB01 molecules to be located in the α1 domain, which covers residue fragments including Pro_15_–Asp_16_–Arg_17_, Ala_40_–Pro_41_–Asn_42_, Gln_54_–Glu_55_–Gly_56_–Gln_57_, and Arg_75_/Gly_75_–Val_76_. In the α2 domain, there is only one distinct fragment, i.e., Leu_103_–Gly_104_–Pro_105_–Asp_106_–Gly_107_–Leu_108_–Leu_109_ between SLA-2-HB01 and SLA-1*0401 molecules (Figure 3B). For SLA-2*HB01 and SLA-3*hs0202, more disordered structural areas are observed in addition to the different areas noted above, also including Asp_39_–Asn_40_–Pro_41_ and Tyr_85_–Asn_86_ in the α1 domain and Thr138–Ala139–Ala140–Gln141, Asp129, Ser132–Trp133–Thr134, and Thr138–Ala139–Arg140–Asn141 in the α2 domain (Figure 3C). Further comparing peptide refolding across the three SLA-I molecules, it was shown that the special refolding state is highly similar within the peptide positions from P1 to P4, and for P9, whereas it was quite different from P5 to P8 (Figure 3D).

**Figure 4:**
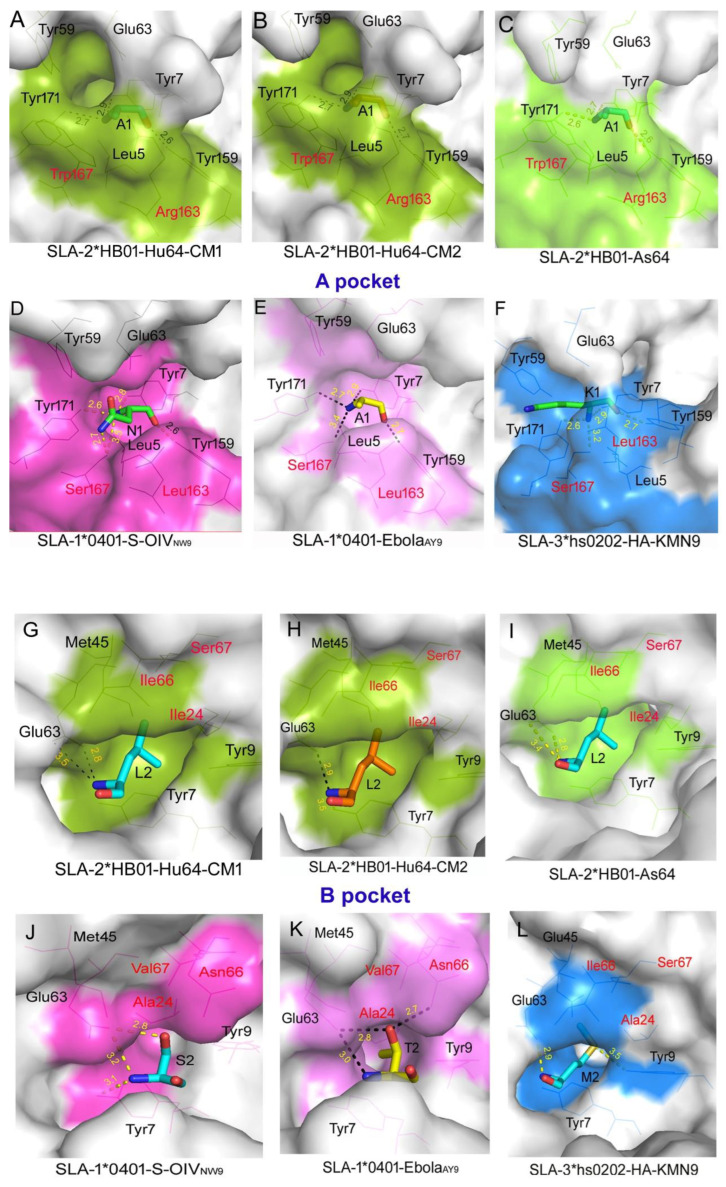
Comparison of pockets A and B in SLA-2*HB01, SLA-1*0401, and SLA-3*hs0202. Pockets are shown as surface representations in white color. The residues comprising the pocket are shown as lines. The residues labeled in black are conserved (no mutation in the above molecules), whereas those in red differ. (**A**–**F**) Pocket A is shown as a surface in SLA-2*HB01–Hu64-CM1, SLA-2*HB01–Hu64-CM2, SLA-2*HB01–As64, SLA-1*0401–S-OIV_NW9_, SLA-1*0401–Ebola_AY9_, and SLA-3*hs0202–HA kmN9, respectively; (**G**–**L**) Pocket B is shown as a surface in SLA-2*HB01–Hu64-CM1, SLA-2*HB01–Hu64-CM2, SLA-2*HB01–As64, SLA-1*0401–S-OIV_NW9_, SLA-1*0401–Ebola_AY9_, and SLA-3*hs0202–HA kmN9, respectively. The dashes indicate the key hydrogen bonds between atoms of the peptides and atoms of the main chain residues. Either black or yellow is used to display the key hydrogen bonds in different background pictures for optimal clarity. Numbers marked in different colors indicate the hydrogen bond difference.

As mentioned above, the conformational differences between Hu64/As64, S-OIV_NW9_/Ebola_AY9_, and HA kmN9 are associated with the variable conformations of the residues that constitute the bottom of the peptide-binding groove of SLA-1, SLA-2, and SLA-3. We conducted further comparison of the pockets of the peptide-binding groove of the three molecules, including pocket A–F. For pocket A (Figure 4A–F), there are eight amino acids, including Leu5, Tyr7, Tyr59, Glu63, Tyr159, Leu163, Ser167, and Tyr171. It was shown that SLA-2*HB01 differs from SLA-1*0401 and SLA-3*hs0202 only at positions 163 and 167, whereas SLA-1*0401 and SLA-3*hs0202 are completely identical. It was shown that pocket A in SLA-1*0401 and SLA-3*hs0202 resembles a wide pocket, whereas SLA-2*HB01 appears to be a narrower, more closed pocket. Further analysis showed that the long chain Arg 163, as well as Arg 62 (not shown in pocket A) and Trp 167 in SLA-2*HB01, block the opening of the A pocket so that it prefers an amino acid with a small side chain at position P1, such as Ala or Gly. Meanwhile, in SLA-1*0401 and SLA-3*hs0202, the short amino acid chains Leu 163 and Ser 167 tend to form a wide pocket, which can more easily accommodate long-chain amino acids at position P1. Furthermore, the Ser 167 with a hydrophilic side chain in SLA-1*0401 and SLA-3*hs0202 can more easily form hydrogen bonds with P1 residues; therefore, the P1 residues in SLA-1*0401 and SLA-3*hs0202 are expected to be more stable. Although differences exist in the three SLA class I molecules, a common feature was discovered: namely, that Tyr 171 and Tyr 7 determine the conformation of the amino terminal of the P1 residue, whereas Tyr 159 determines the conformation of the carboxyl terminal of the P1 residue. Therefore, the refolded direction of the P1 residue in the three SLA class I molecules is wholly consistent. Notably, although slight differences occur between SLA-2*HB01–Hu64-CM1, -CM2, and SLA-2*HB01–As64, the refolded states of the three crystals were almost the same.

For pocket B, as shown in Figure 4G–L, SLA-2*HB01 and SLA-3*hs0202 differ from SLA-1*0401 at positions 24, 66, and 67. The Ile66 of SLA-2*HB01 and SLA-3*hs0202 swings outward from the pocket compared with the Asn66 of SLA-1*0401 (hydrophobic interaction), which results in the hole of pocket B becoming larger. As position 67 changes from being Val to Ser in SLA-2*HB01 and SLA-3*hs0202, pocket B becomes deeper and more easily binds to large side chain amino acids. In addition, the position 66 residue of SLA-2*HB01 and SLA-3*hs0202 changes from being Asn (hydrophilic) to Ile (hydrophobic), which results in pocket B preferring to bind to hydrophobic amino acids, whereas the B pocket of SLA-1*0401 functions in the opposite way. Therefore, in terms of pocket B, SLA-2*HB01 and SLA-3*hs0202 are more similar than SLA-1*0401. Although differences among the three molecules are found in terms of pocket B, some common characteristics also can be discovered, i.e., that Glu 63 can form a hydrogen bond with the amino group of P2 residues and determine its direction of extension. Therefore, the P2 residues of all three molecules all have similar refolded directions. In addition, the structural character of the B pocket in the three SLA class I molecules is similar to that of the HLA-A [49] and HLA-B molecules [50]; specifically, pocket B of HLA-B62 contains residues Met45, Glu63, Ile66, and Ser67, creating a hydrophobic environment, similar to that of the SLA-2*HB01 and SLA-3*hs0202 crystals.

In terms of pocket D, as shown in Figure S2, SLA-2*HB01 differs from SLA-1*0401 and SLA-3*hs0202–HA kmN9 at positions 66, 97, 155, and 156, where the properties of these residues are substantially different. Position 97 changes from being Ser or Asn to Arg in order to compensate for the reduction in the volume of pocket D, which is caused by position 155 changing from being Arg or Gln to Gly, but P3 is more tolerant to hydrophobic amino acids with a long side chain. Meanwhile, comparing with SLA-1*0401, position 156 in SLA-2*HB01 changes from being Arg (positive) to Glu (negative), resulting in a change in the charge properties of the pocket; it should therefore also have a “one-vote veto” function in determining the binding of peptides [31]. In addition, it seems that the P3 (Leu) in three SLA-2*HB01 crystal conformations, including SLA-2*HB01–Hu64-CM1, SLA-2*HB01–Hu64-CM2, and SLA-2*HB01–As64, acts more like a secondary anchor residue, except for the primary P2 and P9 anchor residues [51], because the Leu is buried more deeply within the groove than in the other three SLA-I crystals. In the two molecular asymmetric units of SLA-2*HB01–Hu64, the conformational change of P5 (Thr) results in an obvious effect, whereby the side chain hydroxyl of CM1 undergoes hydrogen bonding interactions with Glu 152, resulting in higher stability of CM1. In addition, although the P5 (Ser) in SLA-2*HB01–As64 could not form a hydrogen bond with Glu 152, van der Waals interactions occurred, which indicates that having a hydrophilic amino acid in P5 in SLA-2*HB01 crystals should favor interaction with Glu 152. In SLA-3*hs0202–HA kmN9, the mutated Trp152 compared to Glu152 in SLA-2*HB01 and SLA-1*0401 is inclined to create a hydrophobic environment, which is not in favor of forming hydrogen bonds with amino acids in P5. In P4, it seems that a TCR docking residue position exists, because in all six SLA-I crystals and conformations, the P4 residues all reach outside of the groove for TCR recognition [52]. However, the P4 (Arg) in SLA-2*HB01–Hu64 and SLA-2*HB01–As64 is higher than that of the other SLA-I crystals, because the Glu 69 in the α1 helix can form a salt bridge with P4 (Arg). P5 residues in SLA-2*HB01 crystals mostly serve as third anchor residues, because P5 (Thr/Ser) is buried in the groove in SLA-2*HB01–Hu64-CM1 and SLA-2*HB01–As64 but not in the unstable SLA-2*HB01–Hu64-CM2. For the SLA-3*hs0202–HA kmN9 crystals, P5 (Gln) also serves as an anchor residue. However, in the two SLA-1*0401 crystals, the P5 residues act more like TCR docking residues. Comparing the D pocket of the PBG for the two SLA-2*HB01 crystals, it seems SLA-2*HB01–Hu64-CM1 bound more strongly than SLA-2*HB01–As64.

For pocket E, as shown in Figure S3, it seems that SLA-2*HB01 differs from SLA-1*0401 only at position 156, which changed from Arg to Glu. This change causes the side chains of Glu152 of the two molecules to have different extensional orientations; for example, the Glu152 in the SLA-2*HB01 molecule is inclined upward to form hydrogen bonds with P7 (Thr), which causes pocket E of SLA-2*HB01 to be less flat than that of SLA-1*0401. In addition, in SLA-2*HB01, it is the P7 (Thr) that occupies the E pocket, whereas in SLA-1*0401, it is the P6 residues, although the peptides are quite different. Overall, in SLA-2*HB01, the peptides form hydrogen bonds more easily than in SLA-1*0401. It should be noted that, in pocket E of SLA-3*hs0202–HA kmN9, there are three residues that differ from those in the SLA-2*HB01 crystals and two residues that differ from the SLA-1*0401 crystals. This difference leads to the dramatic differences in conformation compared with SLA-2*HB01 or SLA-1*0401 such that the E pocket conformation of SLA-3*hs0202–HA kmN9 is not very clearly defined, although in SLA-3*hs0202–HA kmN9, the occupied peptide residue is also P7, which is similar to SLA-2*HB01.

For pocket F, as shown in Figure S4, SLA-2*HB01 differs from SLA-1*0401 at four amino acid sites: 73, 77, 95, and 97. Among them, positions 77 and 97 may play key roles in determining the conformation of the F pocket of the SLA-2*HB01 complex. Unlike pocket B, which prefers to accommodate large ring amino acids, pocket F of SLA-2*HB01 may prefer long side chain residues, because compared with SLA-1*0401, amino acid 77 changes from Gly to a long side chain Asp, whereas position 97 changes from Ser to a long side chain Arg. This causes the entrance of pocket F to become relatively small and bind more tightly. Therefore, pocket F of SLA-2*HB01 prefers binding with Tyr but not Trp, with a long side chain in SLA-1*0401 because pocket F of SLA-2*HB01 is too small to accommodate the indole ring of Trp. Asp 77 and Arg 97 can form multiple hydrogen bonds with P9 (Tyr) in SLA-2*HB01 but not SLA-1*0401 crystals. In addition, Arg 114 can also compensate for this hydrogen bond, which explains why the carboxyl terminal of peptides (Pc) prefer Tyr to Trp. Upon comparison with SLA-3*hs0202–HA kmN9, seven residues are found to differ from SLA-2*HB01 crystals, namely at positions 73, 77, 80, 95, 97, 114, and 116; this may cause stark differences between the pockets of the two crystals. It seems that the F pocket of SLA-3*hs0202–HA kmN9 is inclined to bind to the hydrophobic amino acids of peptides, because three key positions (Asn 77, Asn 97, and Tyr 116) together create a hydrophobic environment. In addition, Phe 95 results in a narrower F pocket compared to that in SLA-2*HB01 crystals. Therefore, the F pocket of SLA-3*hs0202–HA kmN9 is inclined to accommodate a small and hydrophobic amino acid. In summary, the F pocket of SLA-I is conserved, because the conservative Tyr 84 closes the Pc and causes the Pc amino acids to maintain a similar direction of extension. The conformation of the F pocket in SLA-2*HB01 is more similar to that of SLA-1*0401. In addition, the F pocket is larger in SLA-2*HB01 than in SLA-3* hs0202.

### 3.3. Analysis of Potential TCR Contact with CTL Epitopes in Different SLA Class I Crystals

**Figure 5:**
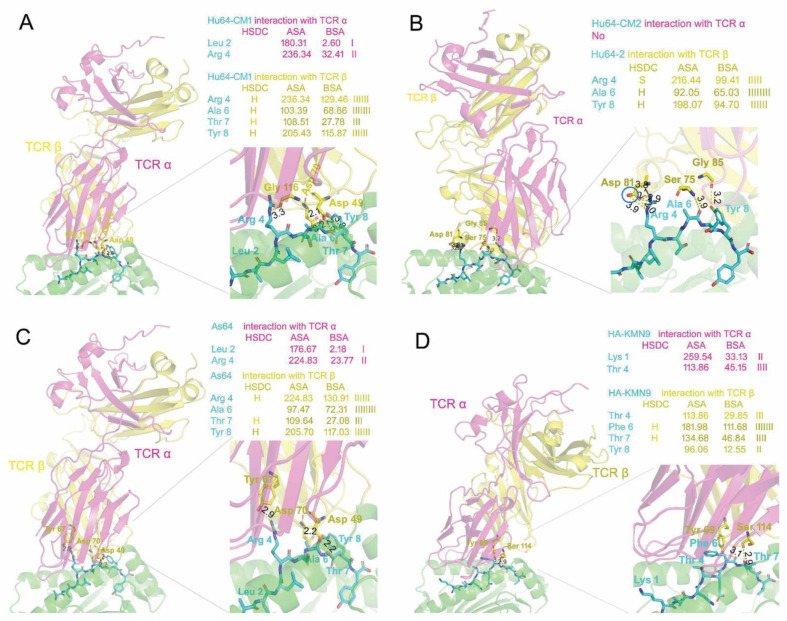
Comparing the conformation of the flexible TCR contact region with different SLA-I molecules. (**A**) The flexible TCR (swine TCR α and β homology modeling based on [54] 1nfd.2.A and 5sws.1.D) contact region with SLA-2*HB01–Hu64-CM1. (**B**) The flexible TCR (swine TCR α and β homology modeling based on 1nfd.2.A and 5swz.3.D) contact region with SLA-2*HB01–Hu64-CM2. The blue circle indicates the carboxyl of Asp 81 in the TCR β chain involved in formation of a salt bridge between Asp 81 in TCR β and Arg 4 in peptide Hu64. (**C**) The flexible TCR (swine TCR α and β homology modeling based on 1nfd.2.A and 5swz.3.D) contact region with SLA-2*HB01–As64. (**D**) The flexible TCR (swine TCR α and β homology modeling based on 1nfd.2. α and 5swz.3.D) contact region with SLA-3*hs0202–HA kmN9. From A to D, the TCR α and β are shown as cartoon models in light magenta and yellow, respectively, whereas the heavy chain of SLA class I complexed with sβ_2_m is shown in green. All of the cartoon models are set as 60 percent transparent. The peptides are shown as sticks in cyan. The number shown in black represents the hydrogen bond distance between the amino acids from TCR and the amino acids from peptides. The threshold for significant value of distance was set as no more than 4.0 Å. In each figure, the left graph shows a general picture of SLA-I complexed with TCR α and β, mainly showing the contact between TCR α and β and peptides bound in the peptide binding groove of SLA-I. The right lower graph indicates the amplified contact area between TCR α and β and peptides. The right upper graph shows the interaction between SLA class I crystals and TCR. HSDC, residues making a hydrogen/disulfide bond, salt bridge, or covalent link; interfacing residues; ASA, accessible surface area, Å²; BSA, buried surface area, Å²; ||||, buried area percentage, one bar per 10%.

Based on the above analysis of the difference in the peptide binding interfaces and pockets between SLA-2*HB01 and other SLA-I molecules, it is essential to examine the potential TCR contact with CTL epitopes in different SLA class I crystals. Using SWISS-MODEL and a protein–protein interaction server, reasonable refolded complexes with swine TCR molecules could be formed in the case of four SLA-I crystals, including SLA-2*HB01–Hu64-CM1, SLA-2*HB01–Hu64-CM2, SLA-2*HB01–As64, and SLA-3*hs0202–HA kmN9; this was determined with reference to the natural structure of mouse MHC class I complexed with mouse TCR molecules [53]. However, another two SLA-I crystals, SLA-1*0401–S-OIVNW9 and SLA-1*0401–EbolaAY9, did not form reasonable refolded complexes based on the present swine TCR database. Overall, the α and β chains first formed homodimers and then made contact with the PBG of the SLA-I crystals to form a new heterodimer, as shown in Figure 5. It was shown that the α chain of TCR made close contact with the α2 domain of PBG, whereas the β chain of TCR was closer to the α1 domain of PBG; this is consistent with the structural characteristics of mouse and human MHC class I complexed with mouse TCR [53,54]. We observe that TCR mainly makes contact with peptides from P4 to P9 in SLA class I crystals. Comparing the differences between different SLA-I crystals that make contact with TCR, it seems that the conformation of TCR in contact with SLA-2*HB01 and SLA-3*hs0202 was similar, except the TCR contact with SLA-3*hs0202 was slightly rotated from right to left compared to in SLA-2*HB01–Hu64-CM1 crystals and SLA-2*HB01–As64 crystals, which causes the TCR α for SLA-3*hs0202 to be inclined to contact the peptides in the groove. Therefore, the TCR α for SLA-3*hs0202 showed more accessible surface area (ASA) and buried surface area (BSA) for peptide amino acids than those for SLA-2*HB01 crystals. In addition, the contact amino acids of TCR (mainly from the β chain) with peptides of SLA-3*hs0202 were completely different from those of SLA-2*HB01. From further analysis and comparison, it seems that Lys 1, which protrudes toward the solvent in SLA-3*hs0202, might cause a total conformation change upon TCR contact, in contrast to the effect of P1 in SLA-2*HB01 upon contact. As for the different SLA-2*HB01 crystals, the overall conformation of TCR was quite similar for SLA-2*HB01–Hu64-CM1 and SLA-2*HB01–As64, as shown in Figure 5A and C. However, the enlarged contact area for TCR and SLA-2*HB01 in the different crystals shows the subtle difference, i.e., the amino acids of TCR that contact peptides were different in the two crystals. As shown in Figure 5A,C, the contact amino acid of TCR with Arg4 of peptide Hu64 in SLA-2*HB01–Hu64-CM1 crystals was Gly116, whereas it was Tyr67 in SLA-2*HB01–As64. In addition, judging from the values for interaction of Hu64-CM1 and As64 with TCR, it seems that ASA and BSA are higher for Hu64-CM1 than As64 upon binding TCR α. However, for TCR β, although Hu64-CM1 forms more hydrogen bonds than As64, it seems the interaction with As64 is fairly stable because As64 can form tighter hydrogen bonds with the corresponding amino acids of TCR β based on the short bond lengths. Just as predicted above, Hu64-CM2 could form a quite different conformation with TCR compared to Hu64-CM1 and As64. It seems that contact between TCR and Hu64-CM2 results in the formation of a linear symmetric body in contrast to TCR contact with Hu64-CM1. As a result of this change, Hu64 is no longer able to come into contact with TCR α in Hu64-CM2 (Figure 5B). In addition, in TCR β, Arg 4 in peptide Hu64 forms a salt bridge with Asp 81, but judging from the bond distance, it could be speculated that the salt bridge is not very strong. Although two hydrogen bonds formed between Ala 6 in peptide Hu64 and Ser 75 in TCR β, and between Tyr 8 in peptide Hu64 and Gly 85 in TCR β, it can be presumed that these bonds are also weak, compared to those in crystal Hu64-CM1 and As64. Furthermore, in Hu64-CM2, the interaction values with TCR β—both BSA and ASA—are lower than those in Hu64-CM1; in Ala6, however, we observed a higher percentage of BSA than in Hu64-CM1. Therefore, it can be speculated that Hu64-CM1 should be more stable in forming a complex of SLA-2*HB01–Hu64-CM1 with TCR. However, as suggested by Figure 1 and Figure 2, if we consider the whole SLA-2*HB01–Hu64 crystal, SLA-2*HB01–As64 might be more stable than SLA-2*HB01–Hu64 in forming a complex of SLA-2*HB01–As64 with TCR.

### 3.4. The Parallel Presentation of Peptide Variants from O and Asia 1 by SLA-2*HB01

**Figure 6:**
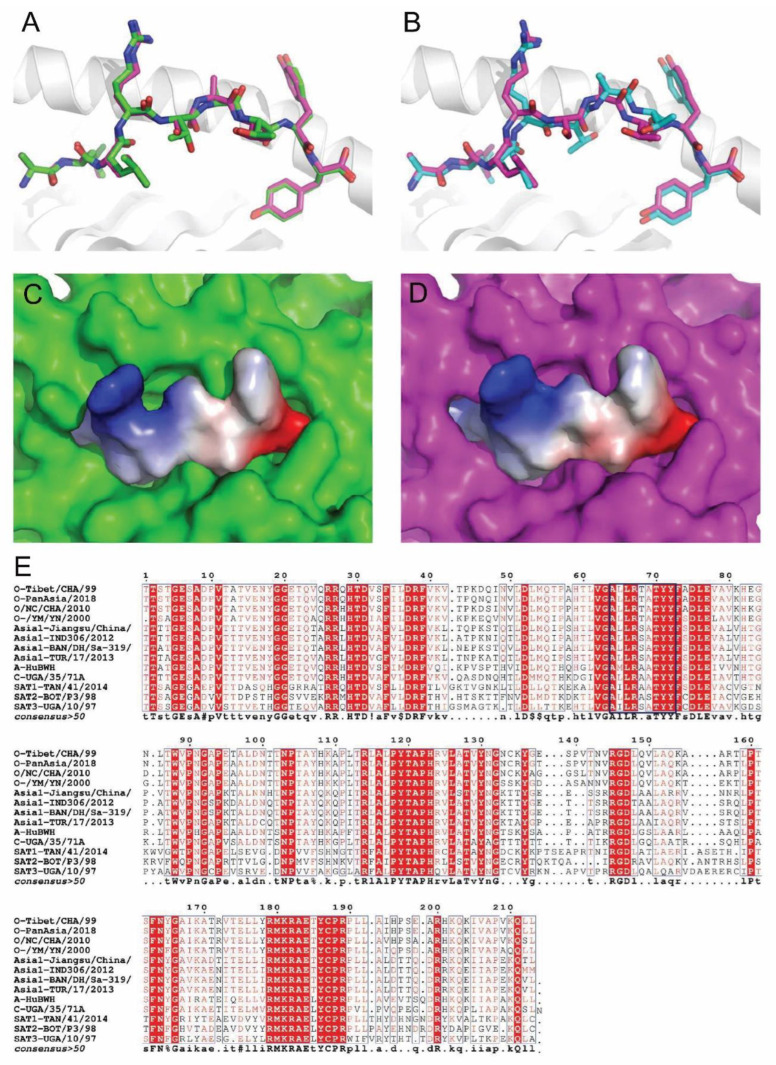
Analysis of the parallel presentation of peptides Hu64 and As64 by SLA-2*HB01, and the peptide conservation. (**A**) The superposition of As64 onto the Hu64 molecule 1 in the asymmetric unit of SLA-2*HB01; (**B**) the superposition of As64 onto the Hu64 molecule 2 in the asymmetric unit of SLA-2*HB01; (**C**) the vacuum electrostatic surface potential of Hu64 molecule 1 in the asymmetric unit; (**D**) the vacuum electrostatic surface potential of As64 exposed out of the peptide binding groove of SLA-2*HB01; (**E**) alignment of VP1 proteins derived from different serotypes of FMDV. The amino acids inside the dark blue quadrant indicate the sequence of peptides Hu64, As64, and Ahu64, which were derived from the O, Asia 1 and A, contrast to those from C, SAT1, SAT2, and SAT3 serotypes of FMDV.

The two peptides Hu64 and As64 are derived from residues 64–72 of the immunodominant antigen VP1 protein of the FMDV O and Asia 1 serotypes, respectively. The two peptides vary according to one residue at the P5 (Thr/Ser) position of the peptides, which indicates that the parallel presentation of peptides by SLA-2*HB01 occurs. However, we must consider how variant or conserved the peptides are when presented in the peptide-binding groove of SLA-2*HB01. In the two structures of Hu64–β_2_m and As64–β_2_m complexed to SLA-2*HB01, we found that the overall structural conformations of SLA-2*HB01–Hu64–β_2_m and SLA-2*HB01–As64–β_2_m are quite similar to each other when presented in the peptide binding groove of SLA-2*HB01. The superposition of the Cα of As64 onto the Hu64 molecule 1 (CM1) (Figure 6A) and molecule 2 (CM2) (Figure 6B) in the asymmetric unit generated root mean square deviation (RMSD) values of 0.285 and 0.694 Å, respectively. The vacuum electrostatic surface potential of Hu64-CM1 (Figure 6C) and As64 (Figure 6D) exposed outside of the peptide binding groove of SLA-2*HB01 also showed similar features.

By aligning the VP1 amino acids of 14 internationally distributed epidemic strains, including four different strains of O serotype (Tibet/CHA/99 (O-HuBHK99), O-PanAsia/2018, O/NC/CHA/2010, and O-/YM/YN/2000), four different strains of Asia 1 serotype (Asia 1-Jiangsu/China/2005, Asia 1-IND306/2012, Asia 1-TUR/17/2013 and Asia 1-BAN/DH/Sa-319/2018), one strain of each from A, C, SAT1, SAT2 and SAT3 serotype (A-HuBWH(1)2009, C-UGA/35/71A, SAT1-TAN/41/2014, SAT2-BOT/P3/98 and SAT3-UGA/10/97, respectively), it was shown that the Hu64 and As64 were positioned at the sites of the 64–72 amino acids of VP1 in O and Asia 1 serotypes, respectively, which are highly conserved in different strains within their corresponding serotypes. In addition, we found that Hu64 and As64 were conserved among different serotypes (Figure 6E). We also found another peptide from A-HuBWH (1) 2009 at the site of 64–72, i.e., AMLRAATYY, known as Ahu64. Compared to Hu64 and As64, there are two amino acids sites that differ for Ahu64: Met at P2 and Ala at P5, with a contrast Leu at P2 and Thr/Ser at P5 in Hu64 and As64. We also attempted the crystallization of SLA-2*HB01-Ahu64: the protein complex can be renatured, but it failed to crystallize (data not shown). It seems that P2 (Met) and P5 (Ala) in peptides may not be in favor of SLA-2-HB01 crystallizing in this assay.

### 3.5. The CTL Activity of Hu64 and As64 Peptides

**Figure 7:**
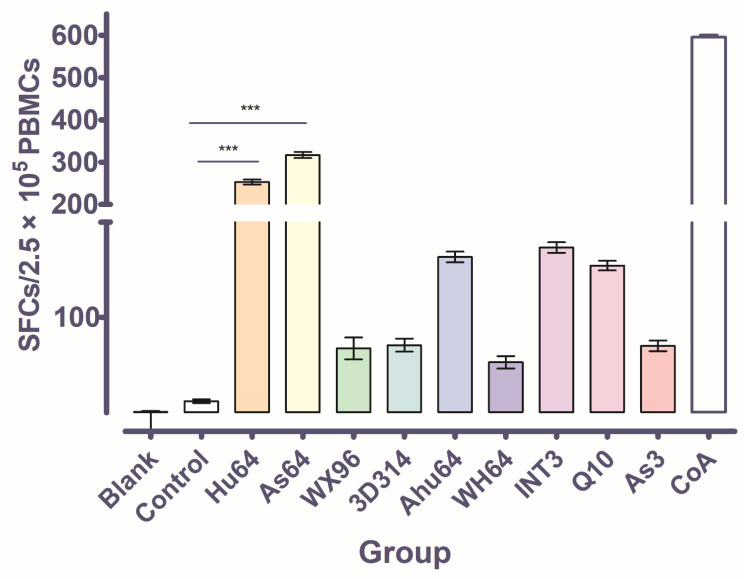
Detection of the peptides that induce CTLs to release IFN-γ by ELISPOT. The break in the y axis indicates a threshold value that can be judged as an effective peptide in the assay. *** indicates the dominant peptides that can induce a superior CTL response. A blank group denotes that there are no peptides detected in the wells of the plates. The control group denotes that the negative peptide control was added to the wells to induce CTLs to release IFN-γ. The CoA group represents the positive control for nonspecific stimulation. The name of each peptide group denotes that a specific peptide was added to the well to induce CTLs to release IFN-γ, and it was detected by ELISPOT.

Earlier studies showed that CTL epitopes can induce specific CD8^+^ T lymphocytes to secrete IFN-γ, which can be detected by ELISPOT [55]. In this assay, the release of cytokine IFN-γ from FMDV-specific CD8^+^ cytotoxic T lymphocytes was induced, under conditions in which the peptides could trigger the recognized CD8^+^ T lymphocytes to produce CTL immunity. In order to obtain the FMDV-specific PBMCs, we adopted an FMDV-challenge assay using three strains of FMDV, including O-HuBHK99, Asia 1-Jiangsu/China/2005, and A-HuBWH(1)2009, which corresponded to peptides designed from three serotypes of FMDV, as shown in Table 1. Before blood sample collection, however, the clinical signs of the infected animals needed to be observed. It was shown that the infected pigs were diseased from the day 3 post-challenge. Although the strains were different, the clinical signs were similar, with vesicles that could be observed on the snout, lips, or on one foot, along with the animal’s body temperature being elevated above 40 °C for 3 successive days. In the ELISPOT detection, ConA (as a positive control) induced the highest IFN-γ release, which indicated the level of IFN-γ-positive cells in total PBMCs in each well. Among the peptide groups, it was shown that As64 can induce the highest IFN-γ release, Hu64 the second, and then INT3, Ahu63, and Q10; meanwhile, the blank control and negative peptide control only displayed a very small number of SFCs. Based on the results of the ELISPOT analysis, the peptides As64 and Hu64 should be the dominant CTL epitopes, as shown in Figure 7.

## 4. Discussion

Hebao is a special breed of pig found in Northeast China. These pigs are inbred and have evolved slowly, with some primary genetic characteristics [36]. Thus, it is important to study the structure of SLA-2 derived from Hebao pigs. Although crystals of SLA-2*HB01 were previously reported, the detailed 3-D structure of them still remains elusive [56].

In this research, we crystallized and analyzed the structure of two SLA-2*HB01–peptide–sβ_2_m complexes associated with two FMDV CTL epitopes, Hu64 and As64. Although there is only one residue difference at P5 between Hu64 and As64, they were derived from two different serotypes of FMDV: the O serotype and the Asia 1 serotype, respectively. Previous studies have shown that the space group of the SLA-2*HB01–Hu64–sβ_2_m and SLA-2*HB01–As64–sβ_2_m crystals belongs to P2_1_2_1_2_1_, with 2.5 Å and 2.4 Å of diffraction resolution, respectively. However, the unit cell parameters of the two crystals were different [35,39]. The electron density analysis revealed that SLA-2*HB01–Hu64–sβ_2_m displayed two different electron density states, whereas SLA-2*HB01–As64–sβ_2_m did not. Therefore, we speculate that the peptide Hu64 might have two different refolding states in the crystals, i.e., there might be two conformations in the crystals. The further refined structure showed that the SLA-2*HB01–Hu64–sβ_2_m crystals do in fact contain two molecules with different conformations in the asymmetric unit, whereas the two molecules in the SLA-2*HB01–As64–sβ_2_m crystals displayed the same conformation in the asymmetric unit. Further analysis showed that conformations 1 (CM1) and 2 (CM2) of the SLA-2*HB01–Hu64–sβ_2_m crystals exist in different folding states, mainly related to P5 residues (Thr), i.e., the P5 (Thr) in CM1, but not CM2, serves as an anchor amino acid. Interestingly, the main difference in the conformation between SLA-2*HB01–Hu64–sβ_2_m and SLA-2*HB01–As64–sβ_2_m was also related to differences in P5, namely a change from Thr to Ser, which also serves as an anchor amino acid. Therefore, the P5 residues in SLA-2*HB01 serve as more flexible amino acids. Because the P5 bound to the D pocket of SLA-2*HB01, we further analyzed the conformation of the three SLA-2*HB01 molecules; surprisingly, we then found that their refolded conformations matched the chemical ‘boat type’ and ‘chair type’. The SLA-2*HB01–Hu64–sβ_2_m-CM1 and SLA-2*HB01–As64–sβ_2_m all displayed ‘chair type’ conformations, whereas the SLA-2*HB01–Hu64–sβ_2_m-CM2 showed a ‘boat type’ conformation. Because the ‘chair’ conformer represents a more stable conformation with low free energy, and the ‘boat type’ represents an unstable conformation with high free energy in chemistry [48,57], we can explain why the conformation of SLA-2*HB01–Hu64–sβ_2_m-CM1 and SLA-2*HB01–As64–sβ_2_m should be more stable than SLA-2*HB01–Hu64–sβ_2_m-CM2. In order to study the structural differences between SLA-2*HB01–Hu64–sβ_2_m-CM1 and SLA-2*HB01–As64–sβ_2_m, we compared the hydrogen bonds and van der Waals interactions for peptides Hu64 and As64 bound to SLA-2*HB01–Hu64–sβ_2_m-CM1 and SLA-2*HB01–As64–sβ_2_m. We found that the main difference existed in the P5 residue, because the P5 (Thr) in Hu64 can form hydrogen bonds with the residue 152 of SLA-2*HB01–Hu64–sβ_2_m-CM1, whereas the P5 (Ser) in As64 cannot form hydrogen bonds with any residues of SLA-2*HB01–As64–sβ_2_m. However, SLA-2*HB01–As64–sβ_2_m can form more van der Waals interactions than SLA-2*HB01–Hu64–sβ_2_m-CM1. In order to further elucidate the complex differences between the three conformations of the SLA-2*HB01 crystals, we analyzed the structure of the pockets associated with each amino acid of the peptide interaction with the corresponding amino acids located on the heavy chain SLA-2*HB01. It was shown that the middle pocket D, where P3 to P5 are located, displayed the most distinctions in terms of distance, as was the case in the above description of the P5 (Thr/Ser) interaction. In addition, we speculate that pockets D and E of the Hu64-CM1 crystals should be the most stable, according to the pocket’s bond length, then the As64, and Hu64-CM2, in turn. This speculation was confirmed by the former analysis of the conformations and flexibilities of peptides bound to SLA-2*HB01 molecules presented with electron density. As shown in Figure 1C–E, the middle of the peptide Hu64 in CM1 should be the most stable, whereas the CM2 should be the most unstable and flexible. However, we found that both the N and C termini of the As64 crystals should be more stable in conformation than those of Hu64-CM1, according to the isotropic B factors [58]. We also attempted to refold and crystallize another peptide, Ahu64, which is derived from A-HuBWH (1) 2009 [59], a serotype of FMDV, with SLA-2*HB01 and sβ_2_m, because there are only two different residues in Ahu64: P2 (Met) and P5 (Ala), in contrast to P2 (Leu) and P5 (Thr/Ser), in the Hu64 and As64 peptides, respectively. However, Ahu64 could not be crystallized with SLA-2*HB01 and sβ_2_m, although it was refolded (data not shown). It can therefore be deduced that the inclusion of P2 (Met) and a hydrophobic Ala in P5 do not favor crystallization in this assay.

Similar to other human HLA class I crystal structures [60,61,62], the total structures of SLA-1, SLA-2, and SLA-3 were similar. However, a more in-depth comparison and analysis of the structures of the three SLA-I molecules represented by SLA-1*0401, SLA-2*HB01, and SLA-3*hs0202 is required. Sequence comparison of the amino acids of SLA-2*HB01 with those of SLA-1*0401 and SLA-3*hs0202 showed that most of the polymorphic residues are located in the α1 and α2 domains, which form close to the peptide-binding groove (PBG) of the SLA-I molecules. Therefore, the main structural differences should be found in the PBGs of the three molecules; for example, the distinct refolded portions between the SLA-1*0401 and SLA-2*HB01 molecules, and between the SLA-3*hs0202 and SLA-2*HB01 molecules, which are all mainly located in the α1 and α2 domains. These structural differences among the three SLA-I molecules could cause the bound peptides to be refolded differently in the PBGs. The most striking finding was that the peptides positioned from P1 to P4, and also P9, had highly similar refolding states, which were quite different to those positioned from P5 to P8.

Further comparison and analysis of the pockets of three SLA-I molecules revealed associations between five crystals and six conformations. However, significant differences were also found; the A and B pockets located on the amino terminals of the PBGs seem more conserved than the middle D or E pockets, and especially compared with the B pocket. These similarities not only exist among different SLA-I molecules but also with human HLA-A and HLA-B molecules [49,63]. It should be noted that although the amino acids in the E pocket seem highly conserved in SLA-2*HB01 and SLA-1*0401, they are in fact flexible, with the E pocket able to accommodate variable peptide amino acids sites: for example, SLA-2*HB01 and SLA-3*hs0202 accommodate P7, whereas SLA-1*0401 accommodates P6. In addition, some SLA-I molecules, such as SLA-3*hs0202, display nearly invisible E pockets [32]. The F pocket also appears highly conserved because of the conservative Tyr 84, as described in the results. In different SLA-I molecules, each pocket displays special characteristics based on the specificity of the bound peptides. However, the most distinctive pocket is the D pocket. In three SLA-I molecules, positions 66, 97, 155, and 156 were the variable amino acids. Among these, position 156 determines the charge property of the pocket. Therefore, it should also have a “one-vote veto” function in determining the binding of peptides, especially to SLA-1*0401 [31]. Turning to SLA-2*HB01, it seems its pocket D became larger and more open because of amino acid changes at the four positions listed above. In addition, it was discovered that Glu 69 and Glu 152 act as key amino acids in maintaining the conformation of SLA-2*HB01–Hu64-CM1 and SLA-2*HB01–As64. In the D pocket, we found that an amino acid at a common position, i.e., P3, acts as a secondary anchor residue because of its close proximity to the highly conserved Tyr 99. However, it was shown that the P3 (Leu) in SLA-2*HB01 is more deeply buried. We also found that P4 (Arg) reached higher levels than that of other SLA-I crystals because the Glu 169 in the α1 helix can form a salt bridge with P4 (Arg). Therefore, we presume that P4 (Arg) should act as a TCR docking residue, just as the P5 residues served as TCR docking residues in the two SLA-1*0401 crystals [31]. We also found that P5 (Thr/Ser) in the pocket D of SLA-2*HB01–Hu64-CM1 and SLA-2*HB01–As64 served as a third anchor residue, similar to the way in which P5 (Gln) in SLA-3*hs0202–HA kmN9 crystals also act as an anchor residue.

Generally, a flexible amino acid in a peptide has a high probability of serving as a TCR docking residue [47,54]. In order to analyze the TCR docking residues in six SLA-I crystals and conformations, we compared the conformations and flexibilities of peptides bound to different SLA-I molecules by showing their electron cloud density (data not shown). It was shown that the TCR docking residues are different in different SLA-I crystals. In SLA-2*HB01 crystals, the potential TCR docking residues are situated on P4, P6, and P8. In SLA-1*0401 crystals, the potential TCR docking residues are situated on P4, P5, and P7. Meanwhile, in SLA-3*hs0202 crystals, the potential TCR docking residues are situated on P4, P6, and P7. It seems that P4 is the common TCR docking residue. In order to further verify potential CTL epitope residues that might contact TCR in different SLA class I crystals, we utilized SWISS-MODEL and the protein–protein interaction server, with reference to the natural structure of mouse MHC class I complexed with mouse TCR molecules [64,65]; four SLA-I crystals, SLA-2*HB01–Hu64-CM1, SLA-2*HB01–Hu64-CM2, SLA-2*HB01–As64, and SLA-3*hs0202–HA kmN9, were found to form reasonable refolded complexes with swine TCR molecules [66]. The results support our hypothesis that P4, P6, and P8 should be the TCR docking residues in SLA-2*HB01 crystals because they are all more deeply buried in the TCR area than other sites of peptides, and most can form hydrogen bonds with the corresponding residues in TCR. For SLA-3*hs0202 crystals, it was shown that P4, P6, and P7 should be the TCR docking residues, according to the same reason outlined above. In this assay, we also attempted to investigate SLA-1*0401 crystals complexed with swine TCR, but, to date, we have not produced a reasonable structure. This work may be attempted again later, when new swine TCR molecules are supplied. In this assay, we also verified that SLA-2*HB01–Hu64-CM1 should be the most stable in complex with TCR, SLA-2*HB01–As64 the second-most stable, and then SLA-2*HB01–Hu64-CM2; this is consistent with the analyses of pocket interactions and conformations, and of the flexibilities of bound peptides. Regarding the potential of Hu64 and As64 as TCR epitopes, we speculate that As64 might be the more dominant epitope for binding to TCR, more strongly inducing CTL immunity because SLA-2*HB01–Hu64-CM1 and SLA-2*HB01–Hu64-CM2 exist in one crystal as two conformations of crystals. However, further research is needed before a definitive conclusion can be reached.

We undertook classical ELISPOT assays to confirm the CTL stimulation function of the peptides. In ELISPOT, it was shown that As64 and Hu64 could induce PBMCs to secrete IFN-γ, producing much higher ELISPOT values compared to other peptides. This means that they should be the dominant CTL epitopes for inducing CTL immunity [67]. Although INT3, Ahu64, and Q10 also induced relatively high SFC values compared to other peptides, they could not induce sufficiently strong CTL immunity. In addition, we also attempted to crystallize these peptides with SLA-2*HB01 and sβ_2_m. However, none of these peptides could produce ideal crystals. Therefore, both the As64 and Hu64 peptides, which crystallized with SLA-2*HB01 and sβ_2_m, should be the dominant CTL epitopes with the capacity to induce T-cell responses.

It was reported that SLA-2*HB01 retained all eight key amino acids that can bind peptides in human HLA-A2—that is Y7, Y59, Y84, T143, K146, W147, Y159, and Y171, which were all found to be conserved residues in the peptide-binding groove of SLA-2*HB01 in Figure 4, Figures S2–S4 in this article, which is completely consistent with previously reported results [36]. In addition, it was also reported that SLA-2*HB01 retained 14 of the 19 amino acids in the α1 and α2 domains of HLA-A2 that bind β_2_m and contained three key amino acids—Gln115 (Q), Asp122 (D), and Glu128 (E)—that bind to CD8 molecules [36]. The two peptides, Hu64 and As4, were identified as SLA-2*HB01-restricted CTL epitopes. Concurrently, we calculated the binding affinities of the two peptides Hu64 and As64 with other SLA-2 molecules, and it was shown that both Hu64 and As64 can bind with most of SLA-2 molecules in the NetMHCpan database, with the exception of one SLA-2*YDY that showed weak binding, whereas the other nine SLA-2 molecules all displayed strong binding (Table S1). Thus, considering that both Hu64 and As64 were highly conserved in correspondence with their serotypes, it is anticipated that Hu64 and As64 may act as potential epitope candidates for the development of a universal vaccine for FMDV in the future.

In order to learn more about the crystals of SLA-2*HB01–Hu64–sβ_2_m and SLA-2*HB01–As64–sβ_2_m, we also supplied the refined crystal data, which are shown in the supplementary file in Table S2.

## 5. Conclusions

In conclusion, in this research, we systematically determined the three-dimensional structure of one SLA-2*HB01 molecule in two crystals associated with three conformations and elucidated the structural characteristics of the SLA-2*HB01 crystals through comparison with other SLA-I crystals. We also confirmed the dominant CTL epitope functions of the two peptides Hu64 and As64, which could be used to develop a CTL epitopes vaccine for FMDV.

## Figures and Tables

**Table 1:** The designed peptides derived from three serotypes of FMDV.

Name	Sequence	Antigen	Position	%Rank	IC_50_ (nM)	Serotype
Q10	LLRTATYYF	VP1	VP1-65–73	0.422	520.43	O
INT3	ILNNIYVLY	3D	3D-308–316	0.559	118.39	Asia I
AS3	TVYNGTSKY	VP1	VP1-127–135	0.05	34.94	A
Hu64	ALLRTATYY	VP1	VP1-64–72	0.538	520.43	O
As64	ALLRSATYY	VP1	VP1-64–72	0.534	155.48	Asia I
WX96	ALNNHTNPTAY	VP1	VP1-96–106	15	2059.23	Asia I
WH64	MLRAATYYF	VP1	VP1-65–73	0.439	430.49	A
3D314	VLYALRRHY	3D	3D-314–322	0.548	132.74	A
AHu64	AMLRAATYY	VP1	VP1-64–72	0.603	73.58	A

Note: rank value is represented by the binding level for peptides, whereas IC_50_ is represented by the affinity between peptides and SLA-2 molecules, according to previous reports [38]. The affinity and rank values were predicted according to automatic calculation on netMHCpan 2.8 (http://www.cbs.dtu.dk/services/NetMHCpan/, accessed on 9 May 2014) when a VP1 or 3D protein sequence of an FMDV strain was input as a FASTA file and SLA-2*HB01 was selected.

**Table 2:** The schedule for virus challenge of pigs according to group.

Group	Numbers	Inoculum	Dose
Control	3	PBS	1 mL
O	3	Tibet/CHA/99 (HuBHK99)	1 mL/10^4^ TCID50
A	3	A-HuBWH (1) 2009	1 mL/10^4^ TCID50
Asia 1	3	1/Jiangsu/China/2005	1 mL/10^4^ TCID50

**Table 3:** Peptide hydrogen bonds and van der Waals interactions in the SLA-2*HB01 heavy chain.

Complex	Peptide		Hydrogen Bond Partner		Distance (Å) ^b^	Van der Waals Contacts Residues
	Residue	Atom	Residue	Atom		
SLA-2*HB01–Hu64 ^a^	P1Ala	N	Tyr171	OH	2.7	Leu5, Tyr7, Glu63, Arg163, Trp167, Tyr171, Tyr159
		N	Tyr7	OH	2.9	
		O	Tyr159	OH	2.6	
	P2Leu	N	Glu63	OE1	2.8	Tyr7, Tyr9, Met45, Glu63, Ile66, Ser67, Tyr99, Tyr159
	P3Leu	N	Tyr99	OH	3.2	Ile66, Tyr99, Glu156, Tyr159
	P4Arg	NH1	Glu69	OE1	2.9	Ile66, Glu69
		NH1	Glu69	OE2	2.9	
	P5Thr	OG1	Glu152	OE2	2.8	Arg97, Glu152
	P6Ala	none				Asn73
	P7Thr	OG1	Glu152	OE1	2.2	Asn73, Trp147, Val150, Glu152
	P8Tyr	O	Trp147	NE1	2.8	Asn73, Val76, Asp77, Trp147
	P9Tyr	N	Asp77	OD1	2.7	Tyr74, Asp77, Thr80, Leu81, Tyr84, Arg114, Asp116, Tyr123, Thr143, Lys146
		OH	Tyr74	OH	2.8	
		OH	Asp116	OD1	2.2	
		OH	Arg114	NH2	3.1	
		O	Lys146	NZ	2.9	
		OXT	Tyr84	OH	3.0	
		OXT	Thr143	OG1	2.5	
SLA-2*HB01–As64	P1Ala	N	Tyr171	OH	2.6	Tyr7, Arg62, Glu63, Tyr159, Arg163, Trp167, Tyr171
		N	Tyr7	OH	2.7	
		O	Tyr159	OH	2.6	
	P2Leu	N	Glu63	OE1	2.8	Tyr7, Tyr9, Met45, Glu63, Ile66, Ser67, Tyr99, Tyr159, Arg163
	P3Leu	N	Tyr99	OH	3.3	Ile66, Arg97, Tyr99, Glu156, Tyr159
	P4Arg	NH1	Glu69	OE1	2.6	Ile66, Glu69
		NH1	Glu69	OE2	3.0	
	P5Ser	none				Glu152
	P6Ala	none				Ser5, Thr7, Asn73
	P7Thr	OG1	Glu152	OE1	2.5	Asn73, Asp77, Trp147, Glu152
	P8Tyr	O	Trp147	NE1	3.0	Asn73, Val76, Asp77, Trp147
	P9Tyr	N	Asp77	OD1	2.9	Tyr74, Asp77, Thr80, Leu81, Tyr84, Arg114, Asp116, Tyr123, Thr143, Lys146, Trp147
		OH	Tyr74	OH	2.7	
		OH	Asp116	OD1	2.4	
		OH	Arg114	NH2	3.5	
		O	Lys146	NZ	2.7	
		OXT	Tyr84	OH	2.6	
		OXT	Thr143	OG1	2.7	

^a^ is indicated for the SLA-2*HB01–Hu64–sβ_2_m-CM1 complex. ^b^ only shows the distance (Å) below 3.5 Å. The underlined amino acids are the different amino acids that bind to peptides via van der Waals between the two complexes.

## Data Availability

The datasets presented in this study can be found in online repositories. The names of the repository/repositories and accession number(s) can be found in the article/Supplementary Materials.

## Supplementary Materials

The following supporting information can be downloaded at: https://www.mdpi.com/article/10.3390/cells11244017/s1, Figure S1: Structure-based sequence alignment of SLA-2*HB01 and previously crystallized SLA-1*0401 and SLA-3*hs0202 molecules. Black arrows above the alignment indicate β-strands; cylinders denote α-helices. Residues highlighted in red are absolutely conserved. Residues in black are amino acids whose chemical or physical characteristics completely differ between the two molecules. Residues in light red are amino acids that are similar in terms of their chemical or physical characteristics. Green numbers denote residues that form disulfide bonds. The alignment was generated using the program Multalin (http://multalin.toulouse.inra.fr/multalin/, accessed on 21 December 2018) [68] and drawn with ESPript [69]; Figure S2: Comparison of the structural differences in pocket D among SLA-2*HB01, SLA-1*0401, and SLA-3*hs0202–HA kmN9. Pockets are shown as surface representations in white. The residues comprising the pocket are shown as lines. The residues labeled in black represent conserved (no mutation in above molecules) residues, whereas red or pink is used to represent mutated residues. (A–F) Pocket D is shown as a surface in SLA-2*HB01–Hu64-CM1, SLA-2*HB01–Hu64-CM2, SLA-2*HB01–As64, SLA-1*0401–S-OIVNW9, SLA-1*0401–EbolaAY9, and SLA-3*hs0202–HA kmN9, respectively. The dashes indicate the key hydrogen bonds between the atoms of peptides and the atoms of the residues of the main chains. For clarity, either black or yellow was used to display the key hydrogen bonds in the different background pictures. Numbers marked in different colors indicate the hydrogen bond difference. This is also the case in Figures S3 and S4; Figure S3: Comparison of the structural differences in pocket E between SLA-2*HB01, SLA-1*0401, and SLA-3*hs0202–HA kmN9. Pockets are shown as surface representations in white. The residues comprising the pocket are shown as lines. The residues labeled in black represent conserved (no mutation in above molecules) residues, whereas red or pink is used to represent the sites of mutated residues. (A–F) Pocket E is shown as a surface in SLA-2*HB01–Hu64-CM1, SLA-2*HB01–Hu64-CM2, SLA-2*HB01–As64, SLA-1*0401–S-OIVNW9, SLA-1*0401–EbolaAY9, and SLA-3*hs0202–HA kmN9, respectively; Figure S4. Comparison of the structural differences in pocket F between SLA-2*HB01, SLA-1*0401, and SLA-3*hs0202–HA kmN9. Pockets are shown as surface representations in white. The residues comprising the pocket are shown as lines. The residues labeled in black represent conserved (no mutation in above molecules) residues, whereas red or pink is used to represent the sites of mutated residues. (A–F) Pocket F shown as a surface in SLA-2*HB01–Hu64-CM1, SLA-2*HB01–Hu64-CM2, SLA-2*HB01–As64, SLA-1*0401–S-OIVNW9, SLA-1*0401–EbolaAY9, and SLA-3*hs0202–HA kmN9, respectively. Table S1. Prediction of epitope Hu64 and As64 binding with different SLA-2 molecules. Note: The affinity and rank values were predicted according to automatic calculation on the updated netMHCpan 4.1 (https://services.healthtech.dtu.dk/service.php?NetMHCpan-4.1, accessed on 17 October 2022) when a VP1 protein sequence from O-HuBHK99 and Asia 1-1/Jiangsu/China/2005 serotypes of FMDV was input as a FASTA file, and SLA-2 molecules were selected. The %rank value is represented by the binding level for peptides, the threshold for strong binding peptides is below 0.500, whereas for weak binding peptides it is below 2.0 [38]. Table S2. X-ray diffraction data processing and refinement. RMSD, root mean square deviation. *^a^* Values in parentheses are given for the highest resolution shell.
