# Supplementary material for: The Parallel Presentation of Two Functional CTL Epitopes Derived from the O and Asia 1 Serotypes of Foot-and-Mouth Disease Virus and Swine SLA-2*HB01: Implications for Universal Vaccine Development
Source: Cells. 2022 Dec 12;11(24):4017. doi: 10.3390/cells11244017 (PMC9777387; doi:10.3390/cells11244017)
Supplement: Supplementary file 1 [file cells-11-04017-s001.zip › cells-1919632-supplementary.pdf]

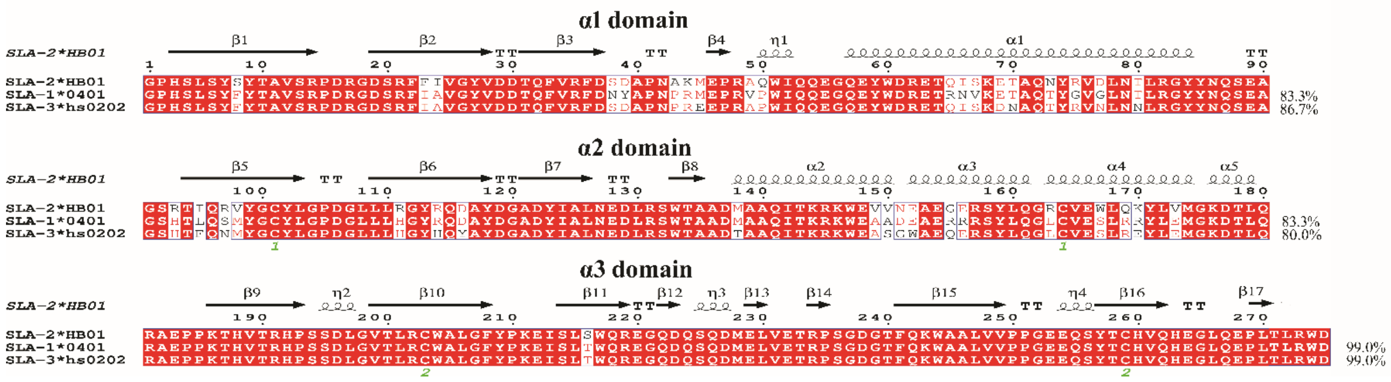

Figure S1: Structure-based sequence alignment of SLA-2*HB01 and previously crystallized SLA-1*0401 and SLA-3*hs0202 molecules. Black arrows above the alignment indicate β-strands; cylinders denote α-helices. Residues highlighted in red are absolutely conserved. Residues in black are amino acids whose chemical or physical characteristics completely differ between the two molecules. Residues in light red are amino acids that are similar in terms of their chemical or physical characteristics. Green numbers denote residues that form disulfide bonds. The alignment was generated using the program Multalin (http://multalin.toulouse.inra.fr/multalin/) [1] and drawn with ESPript [2].

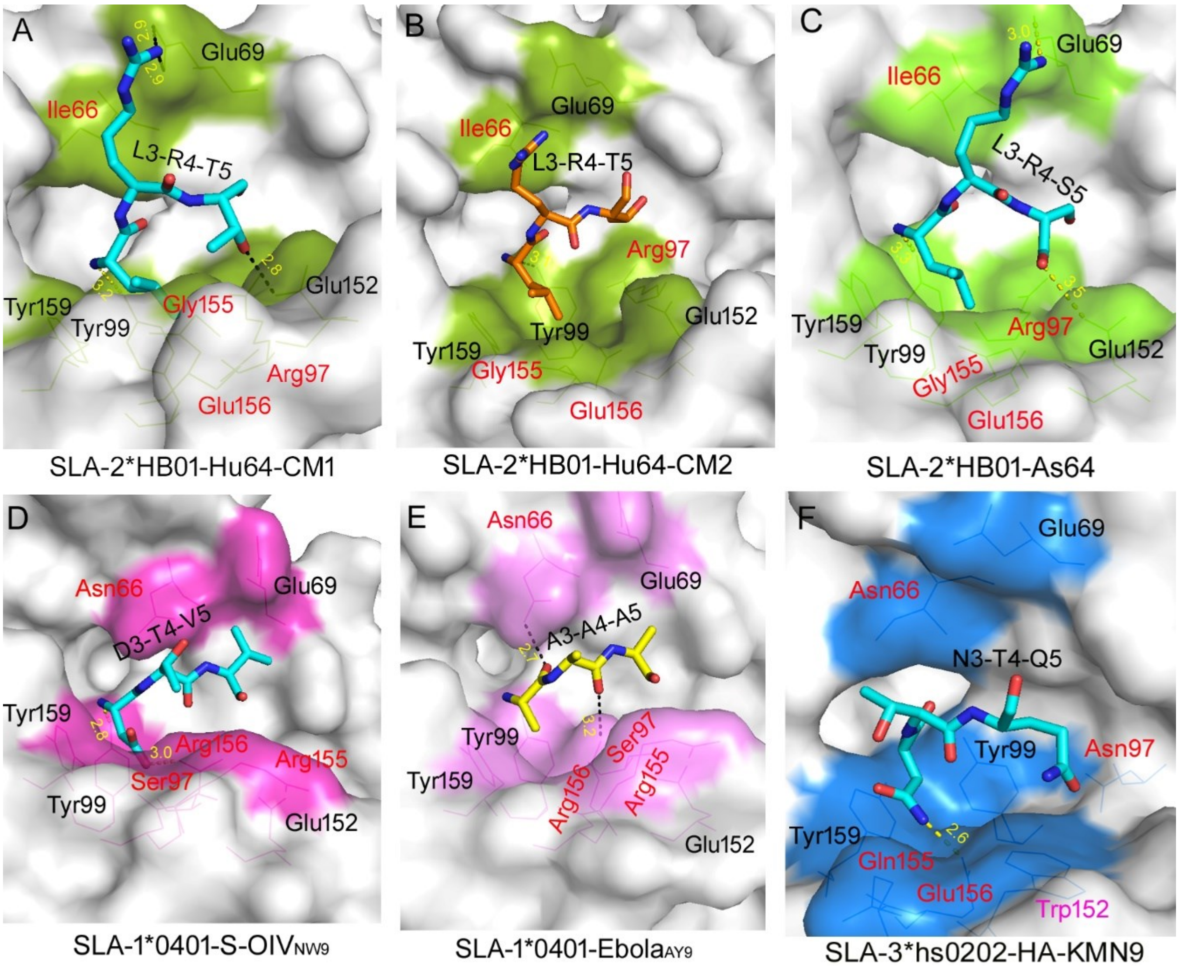

Figure S2: Comparison of the structural differences in pocket D among SLA-2*HB01, SLA-1*0401, and SLA-3*hs0202–HA kmN9. Pockets are shown as surface representations in white. The residues comprising the pocket are shown as lines. The residues labeled in black represent conserved (no mutation in above molecules) residues, while red or pink is used to represent the sites of differed residues. (A-F) Pocket D is shown as a surface in SLA-2*HB01–Hu64-CM1, SLA-2*HB01–Hu64-CM2, SLA-2*HB01–As64, SLA-1*0401–S-OIVNW9, SLA-1*0401–EbolaAY9, and SLA-3*hs0202–HA kmN9, respectively. The dashes indicate the key hydrogen bonds between the atoms of peptides and the atoms of the residues of the main chains. For clarity, either black or yellow was used to display the key hydrogen bonds in the different background pictures. Numbers marked in different colors indicate the hydrogen bond difference. This is also the case in Figure S3-4.

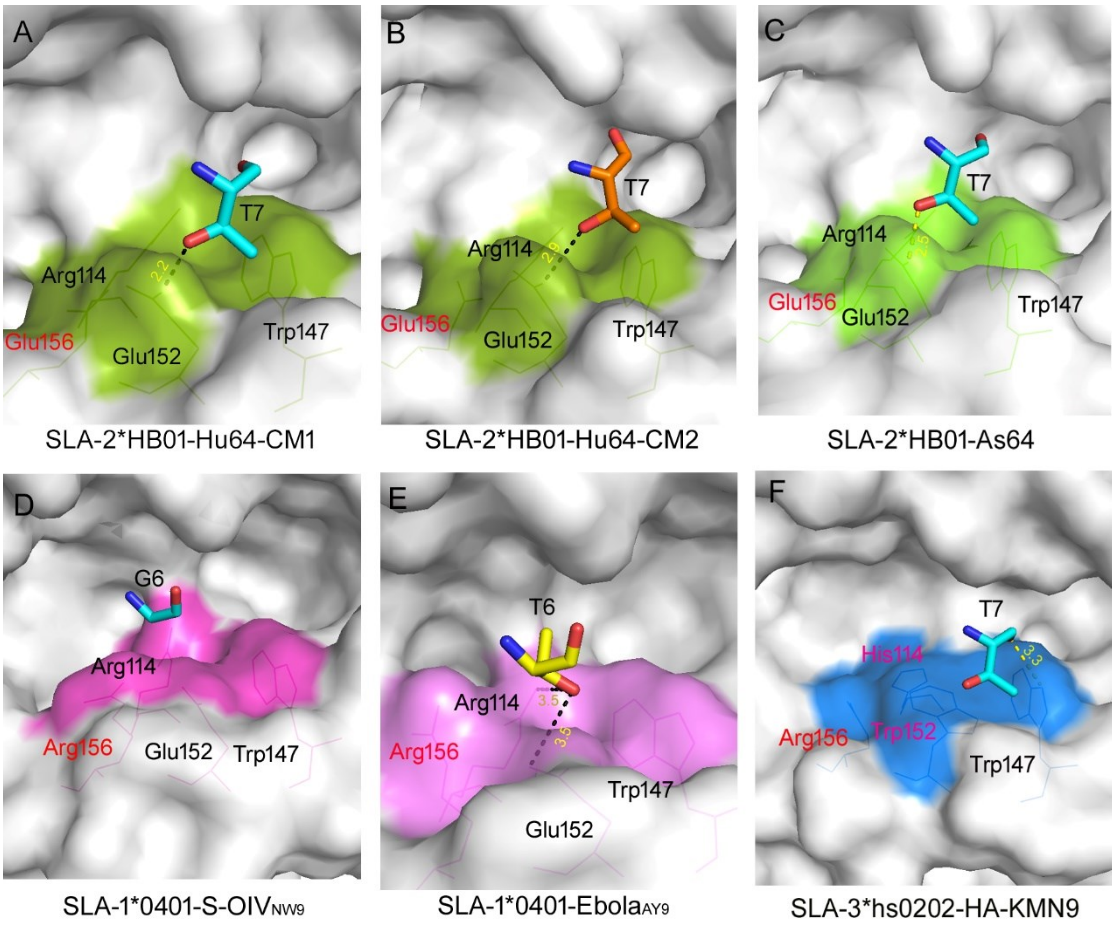

Figure S3: Comparison of the structural differences in pocket E between SLA-2*HB01, SLA-1*0401, and SLA-3*hs0202–HA kmN9. Pockets are shown as surface representations in white. The residues comprising the pocket are shown as lines. The residues labeled in black represent conserved (no mutation in above molecules) residues, while red or pink is used to represent the sites of differed residues. (A-F) Pocket E is shown as a surface in SLA-2*HB01–Hu64-CM1, SLA-2*HB01–Hu64-CM2, SLA-2*HB01–As64, SLA-1*0401–S-OIVNW9, SLA-1*0401–EbolaAY9, and SLA-3*hs0202–HA kmN9, respectively.

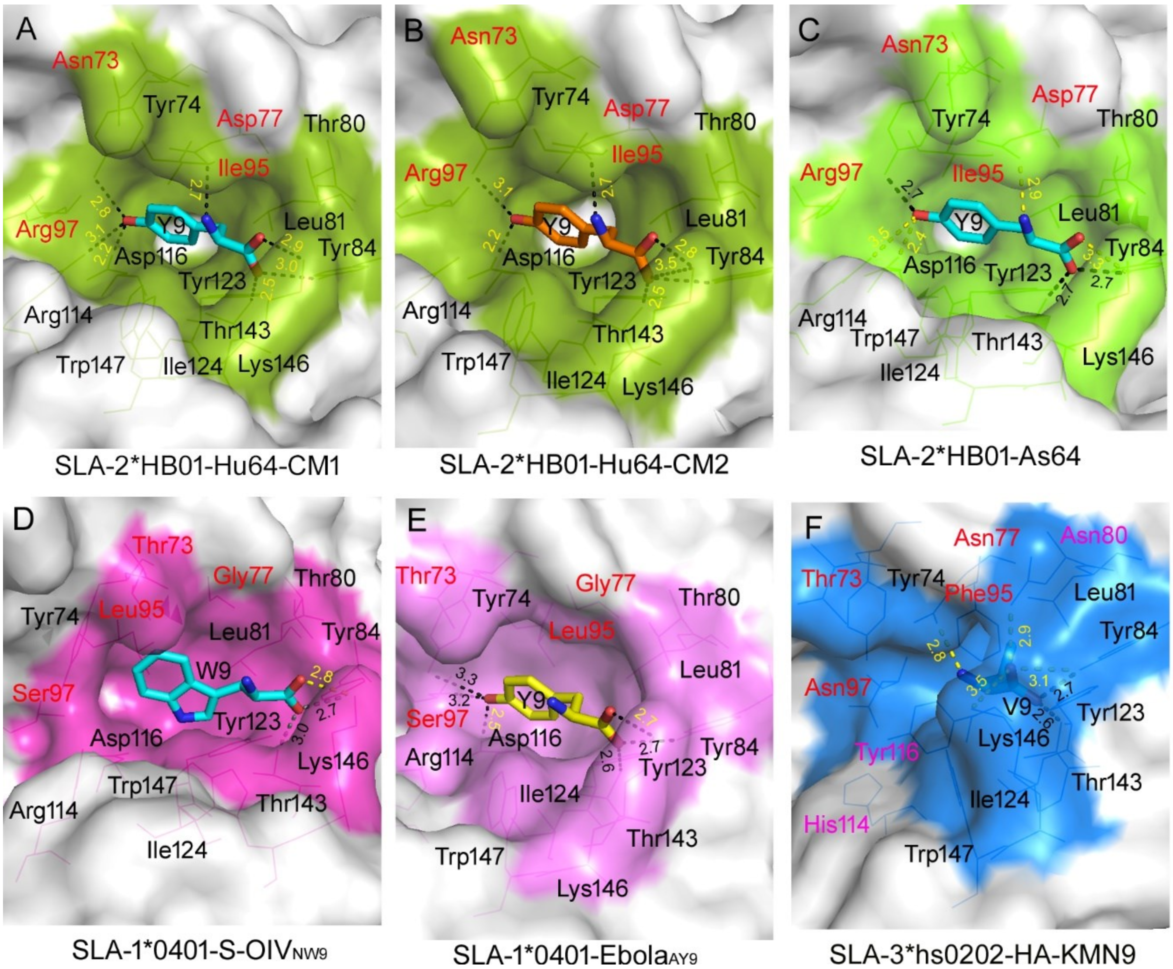

Figure S4. Comparison of the structural differences in pocket F between SLA-2*HB01, SLA-1*0401, and SLA-3*hs0202–HA kmN9. Pockets are shown as surface representations in white. The residues comprising the pocket are shown as lines. The residues labeled in black represent conserved (no mutation in above molecules) residues, while red and pink is used to represent the sites of differed residues. (A-F) Pocket F shown as a surface in SLA-2*HB01–Hu64-CM1, SLA-2*HB01–Hu64-CM2, SLA-2*HB01–As64, SLA-1*0401–S-OIVNW9, SLA-1*0401–EbolaAY9, and SLA-3*hs0202–HA kmN9, respectively.

Tables

Table S1. Prediction of epitope Hu64 and As64 binding with different SLA-2 molecules

SLA-2 Name	SLA-2 GenBank No.	Epitope		%Rank	Binding level
		Sequence	Serotype		
SLA-2*HB01	AB602431	Hu64- ALLRTATYY	O	0.055	Strong
		As64-ALLRSATYY	Asia 1	0.048	Strong
SLA-2*0401	KU754560	Hu64- ALLRTATYY	O	0.423	Strong
		As64-ALLRSATYY	Asia 1	0.385	Strong
SLA-2*0101	KU754559	Hu64- ALLRTATYY	O	0.358	Strong
		As64-ALLRSATYY	Asia 1	0.377	Strong
SLA-2*0102	AB845322	Hu64- ALLRTATYY	O	0.214	Strong
		As64-ALLRSATYY	Asia 1	0.204	Strong
SLA-2*0302	AB847444	Hu64- ALLRTATYY	O	0.269	Strong
		As64-ALLRSATYY	Asia 1	0.280	Strong
SLA-2*1001	AB847446	Hu64- ALLRTATYY	O	0.118	Strong
		As64-ALLRSATYY	Asia 1	0.111	Strong
SLA-2*1002	MH295106	Hu64- ALLRTATYY	O	0.075	Strong
		As64-ALLRSATYY	Asia 1	0.062	Strong
SLA-2*LWH	AB672506	Hu64- ALLRTATYY	O	0.442	Strong
		As64-ALLRSATYY	Asia 1	0.424	Strong
SLA-2*YDL	Submited to NetMHCpan, kept in our lab	Hu64- ALLRTATYY	O	0.122	Strong
		As64-ALLRSATYY	Asia 1	0.103	Strong
SLA-2*YDY	Submited to NetMHCpan, kept in our lab	Hu64- ALLRTATYY	O	0.840	Weak
		As64-ALLRSATYY	Asia 1	0.704	Weak

Note: The affinity and rank values were predicted according to automatic calculation on the updated netMHCpan 4.1 (https://services.healthtech.dtu.dk/service.php?NetMHCpan-4.1) when a VP1 protein sequence from O-HuBHK99 and Asia 1-1/Jiangsu/China/2005 serotypes of FMDV was input as a FASTA file, and SLA-2 molecules were selected. The %rank value is represented by the binding level for peptides, the threshold for Strong binding peptides is below 0.500, while for weak binding peptides is below 2.0 [3].

Table S2. X-ray diffraction data processing and refinement.

Data	Hu64(8GQW)	As64(8GQV)
**Data processing**		
**Space group**	$P2_12_12_1$	$P2_12_12_1$
**Cell parameters**		
**a (Å)**	48.63	48.37
**b (Å)**	98.46	97.75
**c (Å)**	166.23	166.16
**α (°)**	90	90
**β (°)**	90	90
**γ (°)**	90	90
**Resolution range(Å)**	50-2.50(2.59-2.50)[a]	50-2.40(2.49-2.40)
**Total reflections**	171,954	222,792
**Unique reflections**	29,138	31,827
**Completeness (%)**	99.7(99.8)	99.2(97.6)
**R_{sym} (%)**	12.4(63.1)	7.8(31.8)
**I/σ**	13.88(2.92)	21.8(6.0)
**Refinement**		
**R_{work} (%)**	21.3	20.5
**R_{free} (%)**	26.0	24.9
**RMSD**		
**Bond lengths(Å)**	0.006	0.005
**Bond angles (°)**	1.149	1.003
**Ramachandran plot quality**		
**Most favored (%)**	96.28	95.21
**allowed (%)**	3.32	4.12
**Disallowed (%)**	0.40	0.66

RMSD, root mean square deviation.

[a] Values in parentheses are given for the highest resolution shell.

References

1. Corpet, F. Multiple sequence alignment with hierarchical clustering. *Nucleic Acids Res* **1988**, *16*, 10881-10890.
2. Robert, X.; Gouet, P. Deciphering key features in protein structures with the new ENDscript server. *Nucleic Acids Res* **2014**, *42*, W320-324, doi:10.1093/nar/gku316.
3. Gao, F.S.; Zhai, X.X.; Jiang, P.; Zhang, Q.; Gao, H.; Li, Z.B.; Han, Y.; Yang, J.; Zhang, Z.H. Identification of two novel foot-and-mouth disease virus cytotoxic T lymphocyte epitopes that can bind six SLA-I proteins. *Gene* **2018**, *653*, 91-101, doi:10.1016/j.gene.2018.02.025.
